# Risk prediction rules in individuals at risk of rheumatoid arthritis: systematic review and validation using the APIPPRA cohort

**DOI:** 10.1016/j.ero.2026.100228

**Published:** 2026-08-24

**Authors:** Marianna Jasenecova, Gabrielle Harker, Maryam Adas, Sumera Qureshi, Laurence Duquenne, Michelle Wilson, Elizabeth M A Hensor, Paul Emery, Kulveer Mankia, Katie Bechman, Kathryn Steel, Yang Luo, Sam Norton, Andrew P Cope

**Affiliations:** 1 Centre for Rheumatic Diseases, King’s College London, London, UK; 2 Leeds Institute of Rheumatic and Musculoskeletal Medicine, University of Leeds, UK; 3 National Institute for Health and Care Research Leeds Biomedical Research Centre, Leeds, UK; 4 Kennedy Institute of Rheumatology, University of Oxford, Oxford, UK

## Abstract

**Objectives:**

To review risk prediction rules (RPRs) in individuals at risk of rheumatoid arthritis (RA) and to externally validate them using an independent interception trial cohort.

**Methods:**

We performed a systematic literature search of MEDLINE and EMBASE on November 30, 2023, updated on July 10, 2025, to identify studies reporting RA RPRs in individuals at risk of RA. Each RPR was assessed across 4 Prediction Model Risk of Bias Assessment Tool domains: participants, predictors, outcome, and analysis. Eligible RPRs were externally validated by examining risk score distributions by RA progression, and evaluating discrimination, predictive accuracy and calibration.

**Results:**

We identified 25 studies describing 41 RPRs in at-risk individuals. Rules incorporating clinical and serological prognostic factors, with or without imaging, were most common. Internal validation was reported in 23 (56%) RPRs and external validation in only 4 (9%). The discrimination C-statistic ranged from 0.59 to 0.98 during development and was presented as the sole performance measure in 21 (51%) RPRs. External validation of 14 RPRs revealed 4 (29%) with moderate discrimination (C-statistic, 0.6-0.7) and 10 (71%) with poor discrimination (C-statistic, <0.6). Brier scores ranged from 0.20 to 0.24 for 6 RPRs, indicating relatively better overall accuracy, whereas 7 RPRs had poorer overall fit (Brier score ≥0.25). Calibration plots showed reasonable calibration in 2 RPRs, with most demonstrating systematic or partial miscalibration.

**Conclusions:**

Existing RA RPRs demonstrated inconsistent performance when externally validated, underscoring population heterogeneity and the need for robust model development, transparent reporting, and independent external validation before clinical implementation.

**WHAT IS ALREADY KNOWN ON THIS TOPIC**

• Rheumatoid arthritis (RA) risk prediction rules (RPRs) combine clinical, serological, genetic, imaging, and laboratory predictors, reflecting multifactorial nature of RA development.
• Most existing RA RPRs rely on internal validation and report discrimination alone, reducing their transportability across different populations.
• Predictive performance may vary with time horizon, with short-term risk estimates being more precise than long-term predictions.

**WHAT THIS STUDY ADDS**

• Serological markers, particularly rheumatoid factor and anticyclic citrullinated peptide antibodies, alongside clinical features, were the most consistently retained and influential prognostic factors, with imaging evidence of subclinical inflammation further improving predictive performance of existing RA RPRs.
• External validation of 14 RA RPRs in the independent Arthritis Prevention in the Preclinical Phase of RA with Abatacept (APIPPRA) placebo cohort revealed variability in discrimination, miscalibration, and overall fit.

**HOW THIS STUDY MIGHT AFFECT RESEARCH, PRACTICE OR POLICY**

• The RA RPRs evaluated in this study highlighted need for further refinement and external validation in other populations.
• Comprehensive evaluation of model performance, including discrimination, calibration and predictive accuracy, is essential for assessing clinical utility.
• Transparent reporting is essential for RA RPRs.
• Existing RA risk prediction models should be recalibrated where possible to facilitate appropriate use in research and clinical practice.

Alt-text: Unlabelled box dummy alt text

## INTRODUCTION

Rheumatoid arthritis (RA) is a chronic systemic inflammatory disease [1,2] affecting an estimated 18.5 million people worldwide in 2023 [3]. In the UK alone, over 483,000 individuals are living with RA [3]. Individuals with RA are also at increased risk of mental health disorders, including depression and anxiety, and commonly experience comorbidities such as cardiovascular and pulmonary disease, which contribute to increased mortality [4]. Given its high prevalence, progressive nature, associated mental health burden, and societal costs, early identification of individuals at risk of developing RA is critical, highlighting the importance of understanding the disease progression from at-risk state to the disease onset.

Over the past 2 decades, studies of individuals at risk of developing RA, including those without clinical synovitis or diagnosed with undifferentiated arthritis (UA), have provided important insights into the identification of at-risk individuals and key underlying risk factors [5]. A consensus-based approach defined clinically suspect arthralgia (CSA) using clinical features, and the presence of ≥3 CSA parameters increased sensitivity and specificity for progression to RA in individuals at risk of RA without clinical synovitis [6]. A subsequent review of 53 studies highlighted serum antibodies to citrullinated protein antigens (ACPA) positivity, subclinical inflammation on imaging, and clinical features as key independent risk factors [7]. Furthermore, insights into the immunopathogenesis of seropositive RA described disease initiation at lung and other mucosal sites following exposure to environmental agents such as smoking, resulting in autoimmune responses to citrullinated and other posttranslationally modified antigens [8]. Well-established modifiable and nonmodifiable risk factors for RA include lifestyle and host-related factors such as increasing age, female sex, or genetic susceptibility, particularly the presence of human leukocyte antigen (HLA)-DRB1 shared epitope alleles [9]. Despite advances in the understanding of RA pathogenesis, predicting progression from a complex set of prognostic factors to clinically apparent disease among individuals at risk of RA remains a major challenge. Recent collaborative efforts between the European Alliance of Associations for Rheumatology (EULAR) and the American College of Rheumatology (ACR) have led to the development of EULAR/ACR risk stratification criteria for development of RA in the risk stage of arthralgia [10]. These criteria were derived from an analysis of data from multiple international cohorts seeking to establish a consensus approach to risk stratification and improve patient selection for future clinical trials.

Clinical prediction rules (CPRs), which are widely used in health and medical research, offer one approach to address this challenge. CPRs are mathematical tools derived from statistical models and can be presented in multiple formats (point-score system, regression formula, calculator, machine learning model, and among others). They have been widely developed and implemented in areas such as cardiovascular risk, breast cancer prognosis, fracture risk assessment, and type 2 diabetes risk [[11], [12], [13], [14], [15], [16]]. These CPRs commonly incorporate combinations of demographic, clinical and laboratory predictors to estimate disease risk or prognosis. However, most recently reported risk prediction models, including in RA, lack external independent validation [17,18]. External validation of predictive algorithms is essential to evaluate their performance and potential generalizability and transportability to routine clinical practice [19]. Assessing combinations of prognostic factors and estimating absolute risk of developing RA enables more accurate identification and stratification of at-risk individuals and supports timely intervention or targeted monitoring in primary or secondary care.

To this end, we set out to (1) identify RA risk prediction rules (RPRs) developed for individuals at risk of RA, describe prognostic factors used within them, and (2) externally validate these RPRs in an independent at-risk cohort of trial participants recruited from 31 centres across the UK and the Netherlands.

## METHODS

### Systematic review

A systematic literature search was performed via Ovid using MEDLINE and EMBASE databases. The primary search was conducted on November 30, 2023 and included records published up to November 29, 2023. A secondary search was conducted on July 10, 2025 to identify any additional records published since the initial search. Search terms included keywords to describe the at-risk population, outcome of RA, clinical arthritis, inflammatory arthritis, persistent arthritis, and the Ingui search filter [20,21] for finding prognostic prediction studies. Complete search terms used are summarised in Supplementary Figure S1. In the original search, records were deduplicated in Ovid and exported into Rayyan for abstract/title screening. Studies selected for full-text screening were exported to EndNote 20. Reasons for exclusion were recorded via Rayyan.

#### Eligibility criteria

Eligible study designs included prospective and retrospective cohort studies, case-control studies, and combined studies with registry databases. Only studies with full-text articles were included; searches were restricted to humans, English language studies and excluded case reports, letters or historical or retracted articles. This review included studies that developed a new RPR or updated an existing one.

Target populations included adults (≥ 18 years) at risk of developing RA. Participants ‘at risk’ of RA were defined as individuals in the preclinical phases of RA or with UA preceding definite RA (eg, genetic/environmental risk factors, RA-related autoimmunity, arthralgia, or unclassified arthritis) [22,23]. The main outcome was development of RA as defined by the revised 1987 ACR [24] or the 2010 ACR/EULAR classification for RA [25]. An RPR was presented as a score point system, an explicitly stated model regression equation or its coefficients, nomogram, machine learning model, or a calculator.

#### Data extraction and risk of bias assessment

Two reviewers (MJ and GH) independently screened study titles and abstracts, with selection of studies for inclusion in the full-text screening. Both reviewers individually selected eligible studies for inclusion in this review. Any discrepancies were resolved through consensus discussion (AC and SN). Authors of relevant studies were contacted when a prediction model was available upon request.

Data extraction from full-text articles and assessing the risk of bias (RoB) and the applicability of predictive models were conducted using the recommended checklist for Critical Appraisal and data extraction for systematic reviews of prediction modelling studies (CHARMS) [26] and the prediction model risk of bias assessment tool (PROBAST) [27]; a merged CHARMS and PROBAST template was used to facilitate extraction and assessment [28]. The template contained a PROBAST summary with automatic calculation of percentages for each RPR and across the main domains, study characteristics and model characteristics tables, adapted versions of which were included in the Supplementary Material.

#### Synthesis of results

A narrative synthesis was undertaken due to heterogeneity in study characteristics and various aspects of reported RA RPRs.

### External validation

#### Validation cohort

The Arthritis Prevention in the Preclinical Phase of RA with Abatacept (APIPPRA) study was a phase 2b, multicentre, randomised, placebo-controlled clinical trial (EudraCT 2013–003413–18) investigating whether targeting participants at high risk of developing RA with T-cell costimulation modulation with abatacept for 12 months can prevent or delay the onset of disease. Those at high risk of developing RA were characterised by the presence of known risk factors such as inflammatory joint pain, serum ACPA and rheumatoid factor autoantibodies, or high-titre ACPA. A full list of study inclusion and exclusion criteria has been reported previously [29], and participant characteristics are included in the trial publication [30]. The validation cohort used in this work was the placebo subgroup of participants recruited to APIPPRA, where the outcome at the end of predefined periods had been documented. Three- and 5-year outcomes were recorded in the APIPPRA Long-Term Outcome (ALTO) study [31]. HLA-DRB1 alleles were imputed from genotypes of the APIPPRA study participant defined on the Infinium Global Screening Array-24 v2.0 using a multiancestry HLA imputation reference panel [32]. No patients or members of the public were involved in this study.

#### Outcome

In the APIPPRA study, RA was defined as the time to fulfilment of the 2010 ACR/EULAR RA classification criteria or the development of ≥3 swollen joints. Cumulative RA incidence was obtained at 1 and 2 years, whereas 3- and 5-year cumulative incidence were derived from the ALTO study in which the primary endpoint was defined using the APIPPRA outcomes and an additional outcome of time to first treatment with disease-modifying antirheumatic drugs, whichever of the 3 occurred first [31].

#### RPRs performance evaluation

Predictive performance of each RPR was evaluated by examining the distribution of risk prediction scores in the validation cohort stratified by outcome and assessing discrimination, predictive accuracy, and calibration [33,34]. For RPRs specified as regression models, risk scores were computed using a linear predictor, defined as a linear combination of regression coefficients and covariates. For each RPR, predictive performance was evaluated and summarised in an overall assessment. Discrimination, calibration, and predictive accuracy (described in the Supplementary Material p3) were then synthesised separately across all RPRs.

### Statistical analysis

Statistical analyses were performed using open-source R version 4.4.1 (2024-06-14 UCRT) [35]. Missing values were assessed for each RPR. Validation of an RPR was not performed if any predictor had >30% missing data. Missing data patterns were evaluated using the VIM R package and, where applicable, imputed using mice R package. Validation involved examining the regression model’s linear predictor stratified by RA progression or summarising individual risk scores into a total prediction score. Where possible, the corresponding probability of developing RA was estimated for each individual.

Area under the curve values with 95% confidence intervals (CIs) were calculated using the pROC R package. The Brier score was measured as the mean difference between predicted probabilities and observed outcomes across all participants. The standard error of the Brier score was estimated as the variance of the squared errors divided by the sample size, and 95% CIs were derived using a normal approximation. Calibration plots were created using the val.prob.ci.2() function from the CalibrationCurves R package. Risk score plots were generated using the ggplot2 R package when insufficient information was available to convert predicted risk scores into predicted probabilities. The calibration slope and intercept were estimated on the logit scale by regressing the observed outcome on the predicted log-odds.

## RESULTS

### Systematic review

#### Search strategy

**Figure 1:**
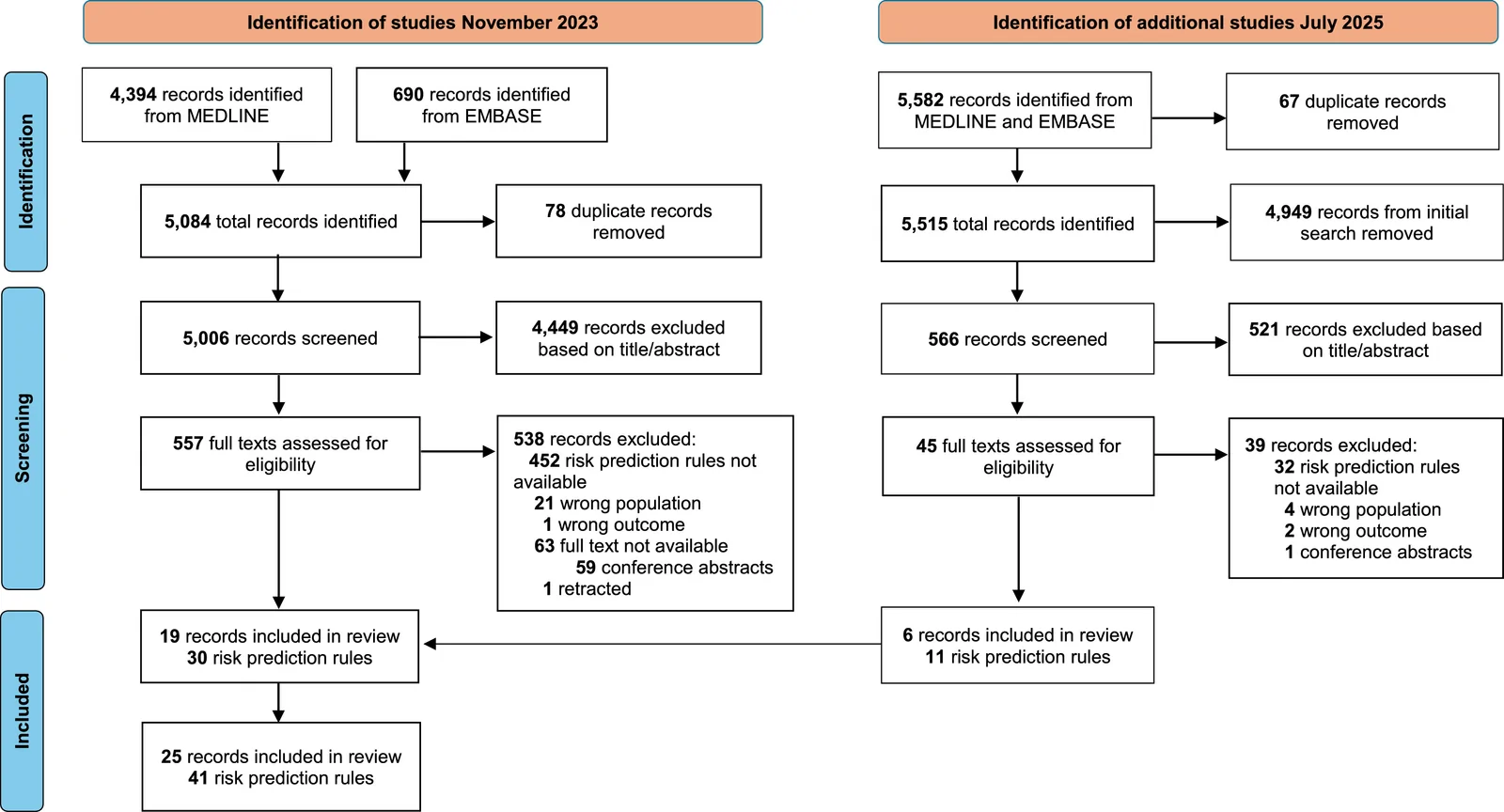
PRISMA flow chart of the studies in the systematic review. PRISMA, Preferred Reporting Items for Systematic reviews and Meta-Analyses.

The primary search identified 5084 articles, of which 5006 records were screened for eligibility. In total, 19 studies with 30 unique RA RPRs were included in the review. A secondary search identified an additional 566 records, of which 45 were selected for full-text screening. When combined, 25 eligible studies reporting 41 RPRs were included in the systematic review (Fig 1) [6,10,[36], [37], [38], [39], [40], [41], [42], [43], [44], [45], [46], [47], [48], [49], [50], [51], [52], [53], [54], [55], [56], [57], [58]].

#### Study characteristics

Of 41 RPRs published between 1998 and 2025, 34 (83%) were developed in Europe, 5 (12%) in Asia and 2 (5%) in the USA. Twenty-seven RPRs (66%) were developed in participants who presented with no clinical synovitis at first visit, 10 (24%) with undifferentiated or inflammatory arthritis with no confirmed RA diagnosis, and 4 (10%) with unknown information about clinical synovitis at inclusion. Twenty-one (51%) RPRs were developed with an RA outcome defined by the 2010 ACR/EULAR or 1987 ACR criteria for RA development, 13 (32%) with an outcome of clinical arthritis in at least 1 swollen joint, 6 (15%) RPRs with a combined outcome, and 1 (2%) with CSA for progression to RA. Thirty-three (80%) RPRs were developed from prospective single or combined cohorts, 3 (7%) from nested case-control, 2 (5%) from retrospective cohort, 2 (5%) from prospective cohorts’ combined registry, and 1 based on a consensus/Delphi process. Key characteristics are summarised in Supplementary Figure S2, and a summary of characteristics of the studies included in the systematic review is provided in Supplementary Table S1.

#### RA risk prediction rule characteristics

**Figure 2:**
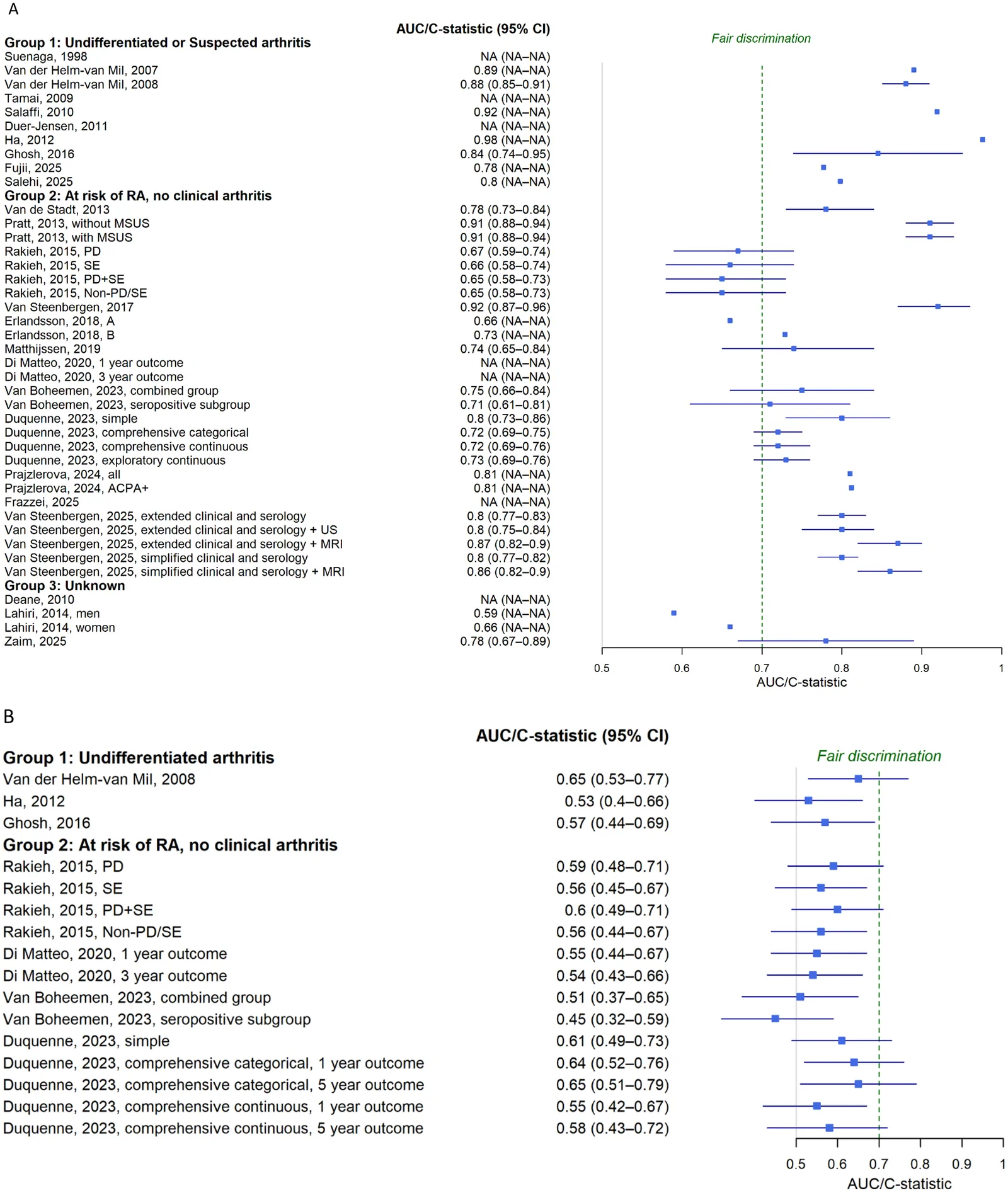
Discrimination of rheumatoid arthritis (RA) risk prediction rules in the development (A) and validation (B) phase.

Sample sizes used for RPR development ranged from 47 to 25,271 individuals, with RA event rates varying between 0.55% and 93%. The number of RA outcomes/events per variable or parameter was estimated to be <10 for 35 of 41 RPRs (85%), between 10 and 20 for 5 of 41 RPRs (12%), and unavailable for 1 study. The number of candidate predictors considered for inclusion in the final RPR ranged from 7 to 204. Predictor selection was based on univariate associations in 28 of 41 RPRs (68%), inclusion of all available predictors in 6 of 41 (15%), prior knowledge in 4 of 41 (10%), and a combination of methods or unclear/missing methods in the remaining 3 of 41 RPRs. Model performance was reported using a discrimination measure in 21 of 41 RPRs (51%), predictive accuracy only in 2 of 41 (5%), and a combination of discrimination with predictive accuracy or calibration in 18 of 41 RPRs (44%) (Supplementary Fig S2). Discrimination C-statistic reported in the development phase ranged from 0.59 to 0.98 (Fig 2A). Internal validation was not reported for 18 of 41 RPRs (44%), whereas 22 of 41 (54%) RPRs used bootstrap, cross-validation, or both methods, and one RPR applied random data split. External validation was reported in 4 of 41 RPRs (9%), whereas 37 of 41 RPRs (90%) had no information on external validation. Handling of missing data was not reported in 19 of 41 RPRs (46%). A detailed summary of RPR characteristics is provided in Supplementary Table S2.

**Figure 3:**
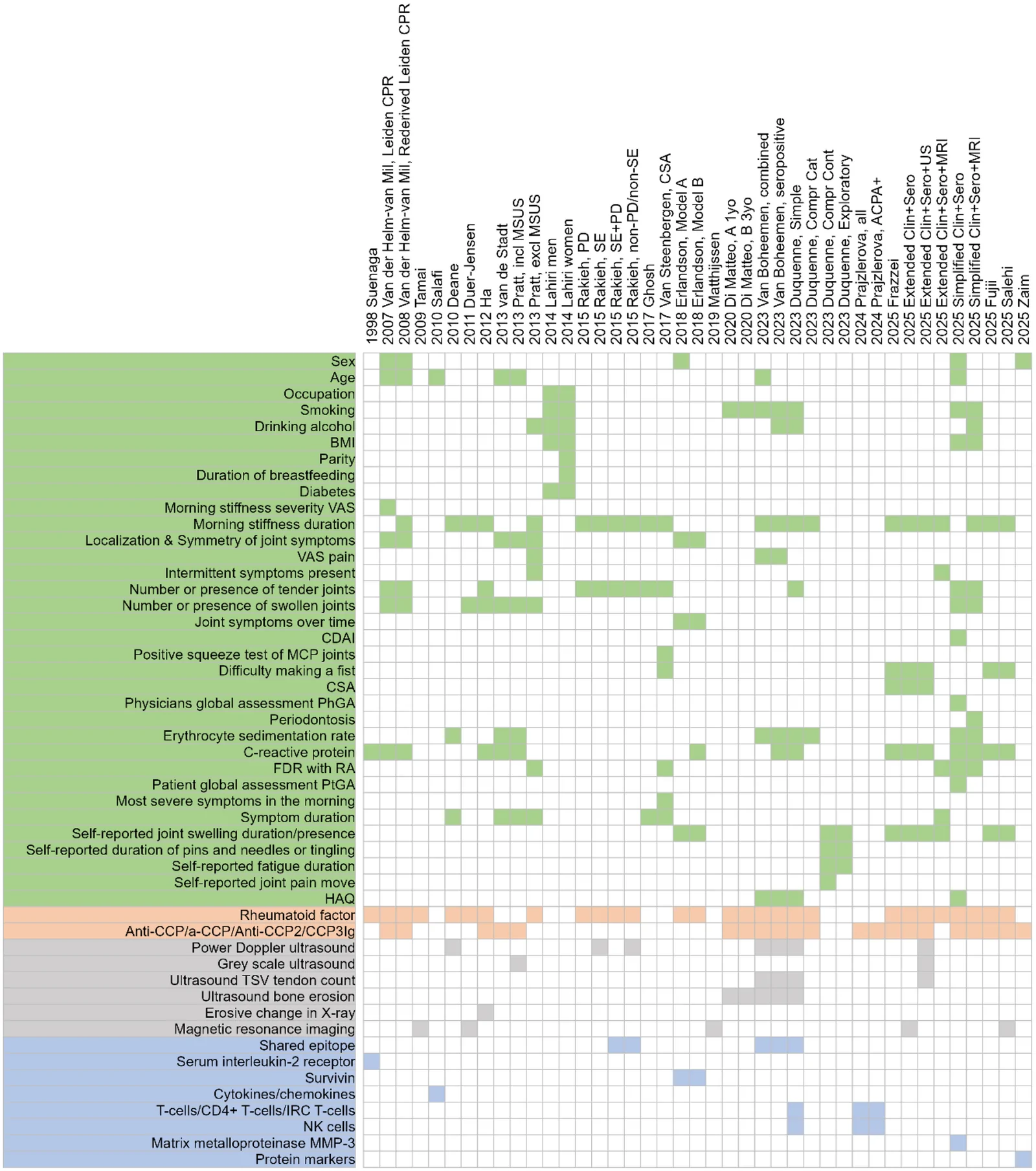
Final prognostic factors in rheumatoid arthritis (RA) risk prediction rules.

**Figure 4:**
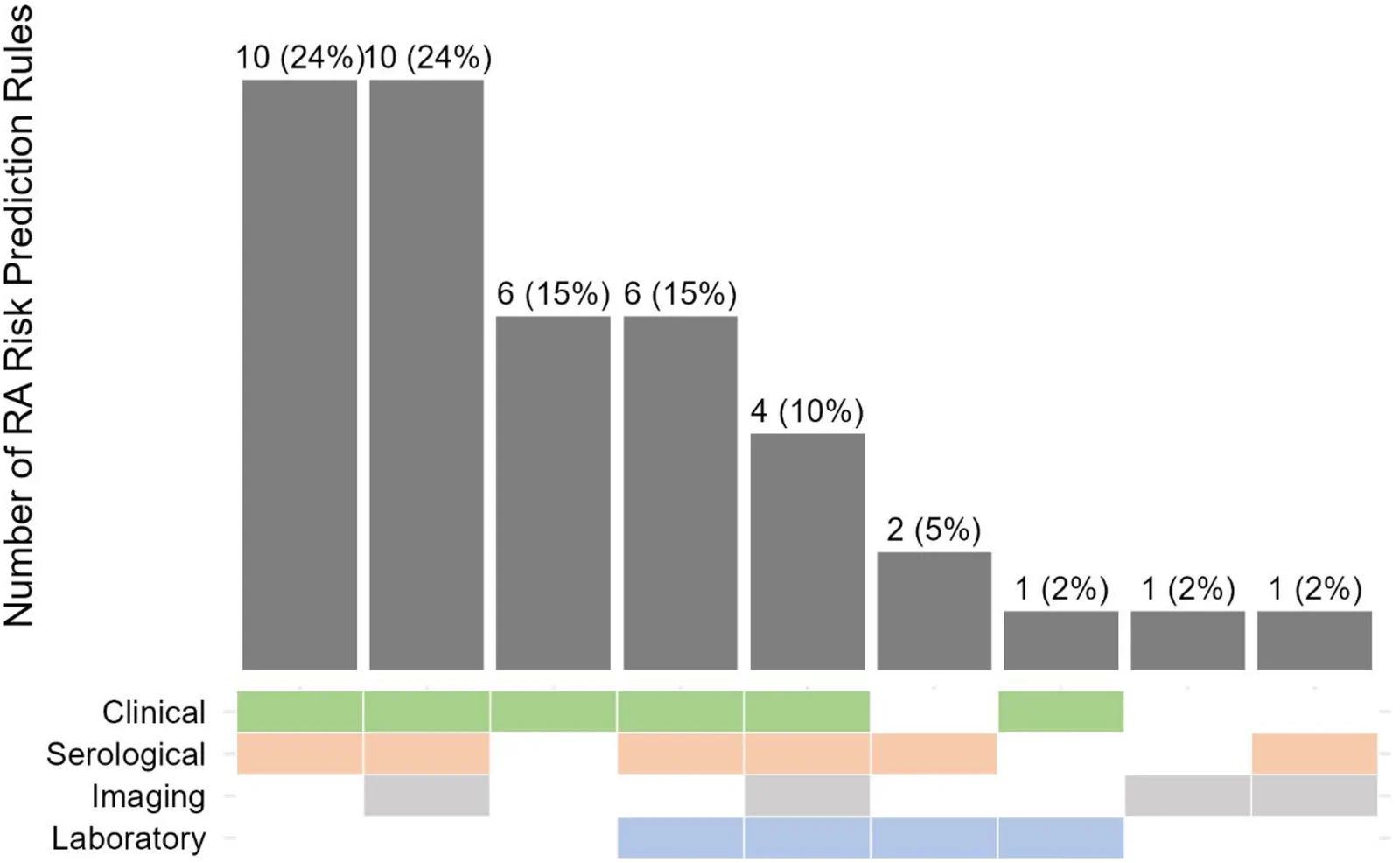
Classification of rheumatoid arthritis (RA) risk prediction rules by final prognostic factors.

Final predictors included in RA RPRs were predominantly serological—ACPA and/or rheumatoid factor utilised 33 times (80%) (Fig 3). Most frequent clinical predictors are summarised in Figure 4 and included morning stiffness duration or severity observed in 23 of 41 RPRs (56%), erythrocyte sedimentation rate (ESR) and/or C-reactive protein (CRP) in 20 of 41 (49%), and tender joint count in 12 of 41 (29%). Laboratory predictors, including a shared epitope and excluding ESR and CRP, were documented in 13 of 41 (41%) RPRs. Most frequently reported demographic and lifestyle factors were smoking status and age in 9 and 8 RPRs, respectively. Imaging predictors were included in 13 of 41 (32%) RA RPRs. When we examined a combination of final prognostic factors, we observed clinical, serological and imaging factors in 10 of 41 RPRs (24%), equally for a combination of clinical and serological factors without imaging. Six RPRs were developed using clinical prognostic factors only or clinical combined with serological and laboratory prognostic factors (Fig 4).

#### RoB and applicability assessment

RoB was assessed across 4 domains: participants, predictors, outcome, and analysis (Supplementary Fig S3A). Overall, high RoB was observed in the development of 37 of 41 RPRs (90%) primarily due to issues in the analysis domain. These included combinations of insufficient sample size, reflected by an event per variable or event per parameter rate of <10; lack of external validation; unfavourable univariate predictor selection method; insufficient reporting of performance evaluation; overfitting; and optimism in model performance. Overall unclear RoB was reported in 4 of 41 RPRs (10%) due to unclear or missing information about predictor inclusion in outcome definitions or outcome assessment without knowledge of predictor information.

Applicability was assessed for participants, predictors, and outcome (Supplementary Fig. S3B). Overall, high applicability concerns were identified in 5 of 41 RPRs (12%), mainly due to discrepancies in outcomes not being explicitly stated as RA, such as arthritis (without ‘rheumatoid’), persistent inflammatory arthritis with a subgroup of RA, polyarthritis, and clinical arthritis. When the outcome was inflammatory arthritis (IA) with over 80% of participants meeting RA classification criteria, the applicability concern was downgraded to low. Unclear concern for applicability was observed in 6 of 41 RPRs (15%) due to unclear participants’ eligibility criteria, insufficient detail on outcome definition, or missing information on musculoskeletal symptoms measured at first visit.

### External validation in the APIPPRA placebo group

#### Validation cohort and missing data

In the APIPPRA study, participants randomised to placebo with no clinical synovitis at baseline and known RA outcome comprised 95 participants after 1 year of follow-up, 92 after 2 years, 86 after 3 years, and 77 after 5 years. The cumulative event rate was 0.31 (29/95) at year 1 and increased to 0.40 (37/92) at 2 years, 0.58 (50/86) at 3 years, and 0.75 (58/77) at 5 years. Main baseline characteristics stratified by RA outcome status at 1 year are presented in Table 1.

**Table 1:** Baseline characteristics of the APIPPRA placebo validation group stratified by rheumatoid arthritis (RA) outcome at 12 months

Characteristic	Non-RA (N = 66)	RA (N = 29)	Total (N = 95)
Age (y)	49.8 (9.6)	51.2 (12.1)	50.2 (10.4)
Sex			
Male	15 (22.7%)	5 (17.2%)	20 (21.1%)
Female	51 (77.3%)	24 (82.8%)	75 (78.9%)
Ethnicity			
White (British)	52 (78.8%)	20 (69.0%)	72 (75.8%)
White (Other)	3 (4.5%)	3 (10.3%)	6 (6.3%)
Mixed	1 (1.5%)	1 (3.4%)	2 (2.1%)
Asian or Asian British	4 (6.1%)	5 (17.2%)	9 (9.5%)
Black or black British	5 (7.6%)	0 (0%)	5 (5.3%)
Other ethnic groups	1 (1.5%)	0 (0%)	1 (1.1%)
Smoking status			
Current	15 (22.7%)	3 (10.3%)	18 (18.9%)
Previous	26 (39.4%)	15 (51.7%)	41 (43.2%)
Never	25 (37.9%)	11 (37.9%)	36 (37.9%)
Anti-CCP level*			
≥3 × ULN (high)	63 (95.5%)	27 (93.1%)	90 (94.7%)
> ULN and ≤3 × ULN	3 (4.5%)	2 (6.9%)	5 (5.3%)
RF and Anti-CCP*			
RF^+^Anti-CCP^+^	53 (80.3%)	27 (93.1%)	80 (84.2%)
RF^−^Anti-CCP^high^	13 (19.7%)	2 (6.9%)	15 (15.8%)
CRP^a^ (mg/L)	6.0 (6.4)	4.1 (3.8)	5.4 (5.7)
Missing	1 (1.5%)	0 (0%)	1 (1.1%)
ESR^a^ (mm/h)	15.9 (14.6)	16.3 (14.6)	16.0 (14.5)
Morning stiffness duration			
None	22 (33.3%)	10 (34.5%)	32 (33.7%)
0-30 min	19 (28.8%)	4 (13.8%)	23 (24.2%)
30 min-1h	10 (15.2%)	7 (24.1%)	17 (17.9%)
1-2 h	7 (10.6%)	4 (13.8%)	11 (11.6%)
2-3 h	4 (6.1%)	1 (3.4%)	5 (5.3%)
3-4 h	0 (0%)	0 (0%)	0 (0%)
4 h-all day	0 (0%)	1 (3.4%)	1 (1.1%)
All day	3 (4.5%)	1 (3.4%)	4 (4.2%)
Missing	1 (1.5%)	1 (3.4%)	2 (2.1%)
TJC44	4.9 (8.2)	6.7 (8.6)	5.4 (8.3)
TJC68	6.2 (11.3)	8.6 (12.0)	6.9 (11.5)

Data are N (%) or mean (±SD).

a Locally measured. APIPPRA, The Arthritis Prevention in the Preclinical Phase of RA with Abatacept; Anti-CCP, Anticyclic citrullinated peptide; CRP, C-reactive protein; ESR, erythrocyte sedimentation rate; RF, rheumatoid factor; TJC, tender joint count; ULN, upper limit normal.

Of 41 RPRs, we validated 14 [[41], [42], [43],^46^,^47^,^51^,^56^], of which 2 were validated for 1- and 5-year outcomes [42]. Remaining studies, including the EULAR/ACR stratification criteria for development of RA, were not validated due to missing predictors exceeding the 30% threshold [6,10,[36], [37], [38], [39], [40],^44^,^45^,^48^,^49^,[52], [53], [54], [55],^57^] or predictors measured in different units with no conversion rate [58] or insufficient information [50]. A summary of missing data for each validated rule and imputation method is described in Supplementary Table S3.

#### Distribution of risk scores

First, we examined the distribution of risk scores, risk groups and linear predictors stratified by outcome status, revealing differences between participants who progressed to RA and those who did not. As expected, the mean risk score or mean linear predictor of the participants who progressed to RA was higher in 93% (13/14) of RPRs compared with participants who did not progress. Models with higher mean scores tended to assign participants to high-risk groups. The proportion of participants assigned to a high-risk group varied across models, ranging from 19% (13/67) to 96% (91/95). A review of risk score distributions revealed that, in 8 point-score systems, no participants achieved the lowest or highest risk score value. Because the RPRs differ in prognostic factors, formats, risk group definitions, and score structures, summary statistics stratified by RA outcome are not pooled across RPRs and are instead presented individually in Supplementary Figures S4A1 to S4P1.

#### Performance evaluation

Across the evaluated RPRs, overall performance varied considerably in terms of discrimination, calibration and predictive accuracy (Table 2, Supplementary Figs S4A2–P2). Discrimination ranged from poor to modest, with C-statistic varying between 0.45 and 0.65, indicating that most models were able to distinguish between participants who developed RA and those who did no better than chance. Four RPRs revealed modest discrimination (C-statistic 0.60-0.65), 9 were poor (C-statistic 0.51-0.59) and 1 had a C-statistic of <0.5 (Table 2, Fig 2B).

**Table 2:** Validation performance in the APIPPRA placebo group

First author, year RPR	Outcome timing	Discrimination^a^	Overall fit^b^	Calibration^c^				Overall assessment^d^
		C-statistic	Brier score	Intercept	Slope	O/E ratio	Plot	
At risk of RA, no clinical synovitis								
Duquenne, 2023 Comprehensive categorical score [42]	1 y	0.64 (0.52-0.76)	0.20 (0.16-0.25)	0.24 (−0.22 to 0.69)	0.79 (0.06-1.51)	1.17 (0.82-1.56)	Figure 5A	Balanced performance with modest discrimination, reasonable calibration, and accuracy; consistent across 1-and 5-y outcomes
Duquenne, 2023 Comprehensive categorical score [42]	5 y	0.65 (0.51-0.79)	0.23 (0.19-0.26)	1.09 (0.54-1.63)	0.82 (0.05-1.58)	1.41 (1.20-1.58)	Suppl. Figure S4N2	Modest discrimination and slope; systematic risk underestimation
Duquenne, 2023 Simple score [42]	1 y	0.61 (0.49-0.73)	0.21 (0.17-0.25)	0.09 (−0.37 to 0.54)	0.64 (−0.02 to 1.29)	1.06 (0.74-1.41)	Suppl. Figure S4L2	Reasonable calibration with modest discrimination
Rakieh, 2015 PD+SE model [47]	2 y	0.60 (0.49-0.71)	0.25 (0.21-0.28)	−0.28 (−0.73 to 0.17)	0.47 (−0.04 to 0.98)	0.87 (0.65-1.11)	Suppl. Figure S4F2	Modest discrimination with calibration slightly conservative
Rakieh, 2015 PD model [47]	2 y	0.59 (0.48-0.71)	0.24 (0.21-0.27)	−0.33 (−0.76 to 0.11)	0.52 (−0.14 to 1.18)	0.85 (0.63-1.07)	Suppl. Figure S4D2	Similar to Rakieh PD+SE model, weaker discrimination, slightly better accuracy, more conservative calibration
Duquenne, 2023 Comprehensive continuous score [42]	5 y	0.58 (0.43-0.72)	0.23 (0.20-0.26)	1.02 (0.47-1.57)	0.30 (−0.36 to 0.96)	1.32 (1.12-1.48)	Suppl. Figure S4P2	Weak discrimination; acceptable predictive accuracy but miscalibrated
Di Matteo, 2020 Model A [43]	1 y	0.55 (0.44-0.67)	0.21 (0.18-0.25)	−0.07 (−0.52 to 0.38)	0.42 (−0.25 to 1.09)	0.96 (0.67- 1.28)	Suppl. Figure S4H2	Accurate on average; poor discrimination
Rakieh, 2015 NonPD/nonSE model [47]	2 y	0.56 (0.44-0.67)	0.25 (0.23-0.28)	−0.36 (−0.79 to 0.07)	0.30 (−0.43 to 1.03)	0.83 (0.62-1.05)	Suppl. Figure S4C2	Poor discrimination and accuracy
Rakieh, 2015 SE model [47]	2 y	0.56 (0.45-0.67)	0.26 (0.22-0.29)	−0.33 (−0.77 to 0.12)	0.31 (−0.23 to 0.84)	0.85 (0.64-1.08)	Suppl. Figure S4E2	Poor overall accuracy with weak calibration slope
Duquenne, 2023 Comprehensive continuous score [42]	1 y	0.55 (0.42-0.67)	0.22 (0.17-0.26)	−0.45 (−1.19 to −0.28)	0.38 (−0.28 to 1.04)	1.06 (0.74-1.41)	Suppl. Figure S4O2	Overpredicts risk; suboptimal slope
Di Matteo, 2020 Model B [43]	3 y	0.54 (0.43-0.66)	0.30 (0.25-0.34)	0.99 (0.54-1.44)	0.32 (−0.29 to 0.93)	1.61 (1.30-1.90)	Suppl. Figure S4I2	Poor discrimination, accuracy and systematic risk underestimation
Van Boheemen, 2023 Combined group [41]^e^	2 y	0.51 (0.37-0.65)	N/A	−0.32 (−1.08 to 0.41)	0.06 (−0.23 to 0.35)	N/A	Suppl. Figure S4J2	Near-random predictions
Van Boheemen, 2023 Seropositive subgroup [41]^e^	2 y	0.45 (0.32-0.59)	N/A	0.03 (−0.88 to 0.94)	−0.10 (−0.42 to 0.21)	N/A	Suppl. Figure S4K2	Minimal predictive value
Undifferentiated/inflammatory arthritis								
Van der Helm-van Mil, 2008 Rederived Leiden CPR [56]	1 y	0.65 (0.53-0.77)	0.25 (0.19-0.30)	−0.83 (−1.35 to −0.32)	0.37 (0.04-0.70)	0.69 (0.49-0.92)	Figure 5B	Modest discrimination; good agreement at lower predicted probabilities, but substantial overprediction at higher predicted probabilities; weak slope
Ghosh, 2016 Simplified prediction formula [46]	1 y	0.57 (0.44-0.69)	0.36 (0.28-0.43)	−2.02 (−2.82 to −1.22)	0.02 (−0.04 to 0.09)	0.65 (0.46-0.87)	Suppl. Figure S4G2	Severe miscalibration; poor accuracy
Ha, 2012 Simplified prediction model [51]	1 y	0.53 (0.40-0.66)	0.56 (0.48-0.65)	−5.03 (−5.63 to −4.43)	0.04 (-0.14 to 0.23)	0.35 (0.25-0.47)	Suppl. Figure S4B2	Poor discrimination; high Brier score; severe miscalibration

Data are estimates with 95% confidence intervals.

a Discrimination was assessed using the C-statistic, equivalent to the area under the curve value for binary outcomes, ranging usually from 0.5 for random guess to 1 for perfect discrimination.

b Predictive accuracy was estimated using the Brier score, where 0 indicates perfect accuracy, 1 for imperfect inaccuracy, and scores <0.25 for more accurate prediction overall.

c Ideal calibration intercept is 0 and ideal calibration slope is 1. An intercept <0 indicates that the model overpredicts risks on average and >0 underpredicts risks on average. A slope <1 indicates that the predictions are too extreme (high risks too high and low risks too low), and >1 means that the predictions are too conservative.

d An overall assessment summarises predictive performance of each RPR based on discrimination, calibration and overall fit.

e A risk score plot was used in place of a calibration plot when insufficient information was available to convert predicted risk scores to predicted probabilities. Calibration intercept and slope were estimated from a regression line in the risk score plot. Brier score and O/E ratio could not be estimated. O/E = ratio of observed-to-expected events based on the model predictions. A value of 1 indicates perfect calibration, a value <1 indicates the model overpredicts risk, and a value >1 indicates underprediction. APIPPRA, The Arthritis Prevention in the Preclinical Phase of RA with Abatacept; CPR, clinical prediction rule; RA, rheumatoid arthritis; RPR, risk prediction rule; N/A, not applicable.

Predictive accuracy, assessed using the Brier score, varied across RPRs and outcome horizons. Six RPRs demonstrated relatively better overall accuracy, with Brier scores ranging from 0.20 to 0.24. In contrast, 7 RPRs exhibited poorer predictive accuracy, with higher Brier scores (≥0.25) where available (Table 2).

Calibration performance differed markedly between RA RPRs. Good calibration, with calibration intercept close to 0 and slopes approaching 1, was demonstrated in 2 RPRs, although CIs were wide (Table 2]). In contrast, 2 RPRs demonstrated substantial miscalibration, characterised by large negative calibration intercepts (<−2) and slopes far <1, indicating systematic overprediction and model overfitting. Three RPRs showed evidence of underprediction (ratio of observed-to-expected [O/E] 1.32-1.61), and 3 showed overprediction (O/E 0.35-0.69), with 95% CIs excluding 1, indicating meaningful miscalibration (Table 2). Eight RPRs had O/E ratios between 0.83 and 1.17, with CIs including 1, indicating good agreement between predicted and observed risk.

An overall assessment of the RPRs is provided in Table 2, integrating evaluations of discrimination, calibration and predictive accuracy and highlighting the relative strengths and weaknesses of each RPR to support comparison across models.

#### Distribution and predictive performance of representative RPRs

**Figure 5:**
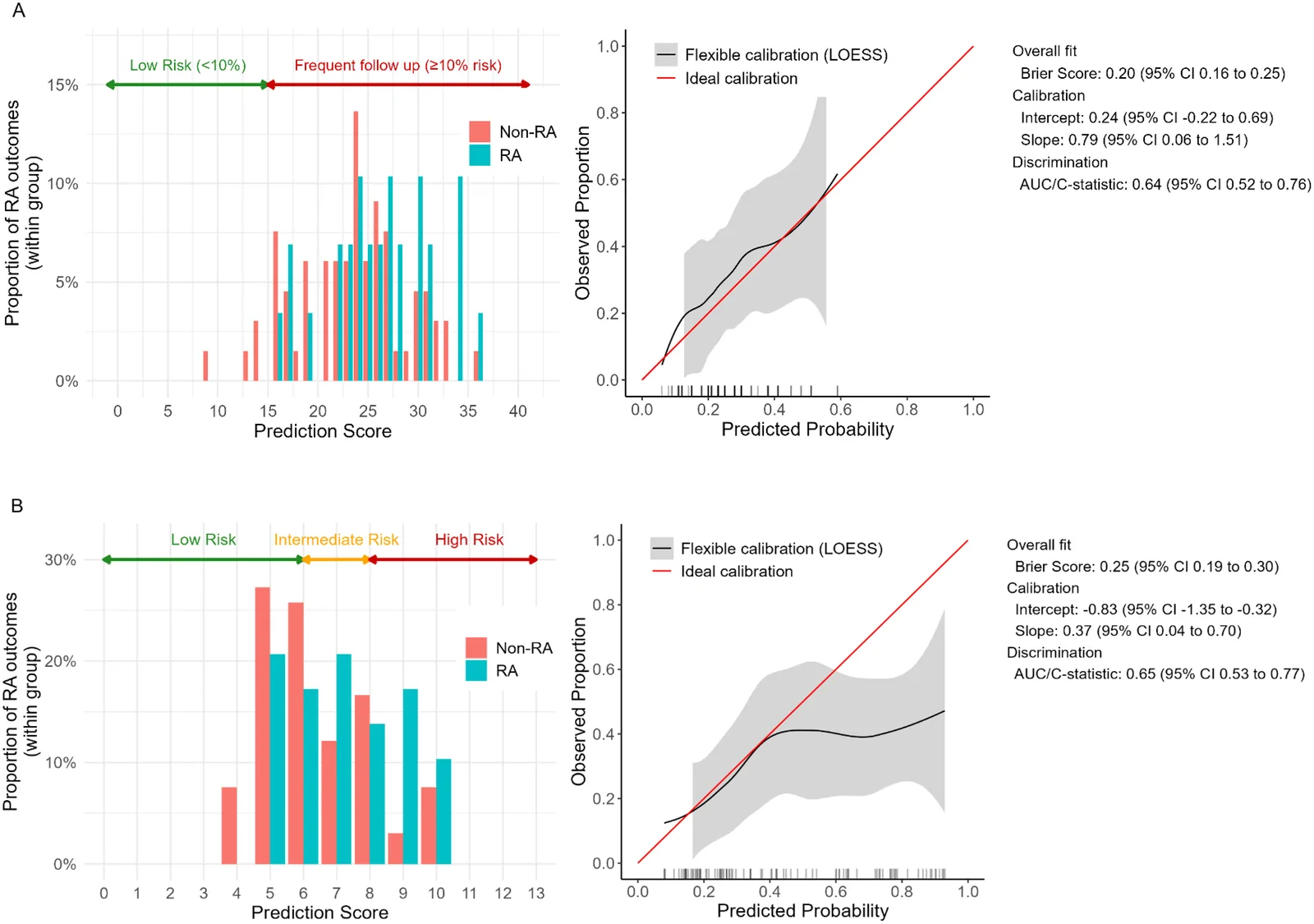
Examples of risk prediction score distribution and rheumatoid arthritis (RA) risk prediction rule performance in the APIPPRA placebo at-risk cohort. APIPPRA, The Arthritis Prevention in the Preclinical Phase of RA with Abatacept.

Among the 14 validated RPRs, the details of the performance of the 2 highest-ranking RPRs are shown for at-risk individuals without clinical synovitis (Fig 5A) and for individuals with UA (Fig 5B), both using 1-year outcomes.

For the Duquenne’s comprehensive categorical scores (Fig 5A left), predicted risk scores ranged from 9 to 36 (mean 24.3, SD 5.6). RA progressors had higher mean scores than nonprogressors (26.2 vs 23.5), and 91 of 95 (96%) participants scored above the threshold of 15 (≥10% risk) for frequent follow-up, as defined by original authors.

For Van der Helm-van Mil’s Rederived Leiden CPR (Fig 5B left), predicted risk scores ranged from 4 to 10 (mean 6.6, SD 1.7). RA progressors had higher mean scores than nonprogressors (6.8 vs 5.7), with 44 of 95 (46%) participants in a low-risk group (score ≤6), 27 of 95 (28%) in an intermediate-risk group (between 6 and 8), and 24 of 95 (25%) in a high-risk group (score ≥8), as defined by original authors.

## DISCUSSION

Although RA evolves as a continuous biological process [59], its multifactorial nature allows disease progression to be captured at identifiable stages, allowing risk to be assessed before clinical onset. Initial assessments in at-risk cohorts establish baseline measurements for participants, facilitating risk stratification, although most prediction models do not fully account for changes over time. The presence, weight, and timing of multiple measurable risk factors are central to determining an individual’s likelihood of developing the disease. These factors are captured in RA risk prediction algorithms, highlighting the importance of comprehensive risk stratification in at-risk individuals, such as the EULAR/ACR stratification criteria for development of RA [10], which were also included in our review.

In this study, we first identified 41 RPRs developed for individuals at risk of RA and summarised the prognostic factors used in their development. We then externally validated 14 RPRs in an independent multicentre cohort of placebo trial participants. Across existing RA RPRs, prognostic factors consistently aligned with biological processes implicated in RA development, spanning systemic autoimmunity, genetic susceptibility, emerging joint symptoms, and imaging evidence of subclinical synovitis. Serological markers, particularly RF and anticyclic citrullinated peptide (anti-CCP) antibodies, were the most consistently retained prognostic factors in existing RPRs and were strongly associated with progression to RA (Fig 3). These autoantibodies reflect early immune dysregulation, characterised by adaptive immune activation and loss of tolerance, which often precedes and likely drives local joint inflammation. Importantly, the presence of RF and/or anti-CCP antibodies has been shown to predict progression to clinically apparent RA in individuals at risk [60,61]. Certain clinical features, including symptom duration, prolonged early morning stiffness and tender joint count, also demonstrated escalation of immune-mediated inflammatory activity within joints and periarticular tissues, rather than symptom burden alone, signalling progression to RA. Imaging findings further anchor this process at the joint and tissue level. In the validation cohort, imaging assessments were restricted to ultrasound-derived measures of subclinical inflammation. Ultrasound features such as power Doppler signal, tenosynovitis, and detected erosions further enhanced RPR performance, reinforcing the role of objectively measured active synovial and peri-synovial inflammation in early RA pathogenesis [62]. Recognising these biological signals before synovitis emerges is central to identifying individuals at the highest risk and informing timely intervention. Although ultrasound is widely available and clinically practical, magnetic resonance imaging scans may provide greater sensitivity for detecting early synovitis, tenosynovitis and structural change; future validation studies could assess whether incorporating magnetic resonance imaging scans further refines risk stratification.

External validation of the 14 RPRs in the APIPPRA placebo cohort revealed that discrimination ranged from poor to moderate, whereas calibration and predictive accuracy varied across RPRs. These results provide additional evidence on the generalizability of these in the population at increased risk of RA. Individual RPRs varied in the weights assigned to each prognostic factor, reflecting differences in derivation cohorts, outcome definitions, and available assessments. Among the RPRs validated in this study, Duquenne’s comprehensive categorical score and the simple score at 1 year, developed for individuals at risk of RA, ranked highest in predictive performance [42] (Table 2). Concerns that the IA outcome did not fully correspond to the RA outcome were partly mitigated by the original authors’ report that 91.9% of IA progressors also met the RA classification criteria at the time of IA diagnosis in the development phase. Among RPRs developed in individuals with UA, Van der Helm-van Mil’s rederived Leiden CPR [56] demonstrated comparatively stronger overall predictive performance than other RPRs (Table 2).

Duquenne’s comprehensive categorical score incorporated clinical, serological, genetic, patient-reported and imaging variables, with RF, anti-CCP and presence of shared epitope contributing most strongly during development. Despite its robust design and relatively stronger prognostic contribution for short-term outcome, the comprehensive categorical 5-year score underestimated absolute risk in our cohort. However, differences in case mix between development (arthralgia with ACPA positive and irrespective of RF) and validation populations (arthralgia with ACPA and RF positive or RF negative and ACPA high) may partly explain the discrepancy between predicted and observed risks, and therefore the observed miscalibration should be interpreted with caution. In Duquenne’s simple score, all APIPPRA participants who progressed to RA within 12 months were classified as high risk (score ≥18; ≥10% risk), demonstrating excellent sensitivity. However, 89% of high-risk individuals did not develop RA within this period. This does not account for progression beyond 12 months and reflects that the threshold was intended to guide referral to secondary care. In practice, such referrals allow close monitoring for disease development, even though many participants may not develop RA in the short term. Duquenne’s comprehensive categorical score based on 1-year outcomes showed better discrimination and predictive accuracy metrics than Duquenne’s simple score, and both scores demonstrated reasonable calibration. Given that the simple score is easier to implement in primary or secondary care with respect to resources, time and practicality, the choice between simple and more comprehensive RPRs may require careful consideration. It also suggests that the predictive ability of risk scores may be stronger over shorter time horizons than longer ones [63].

The rederived Leiden CPR, developed for patients with UA, included clinical and serological prognostic factors and demonstrated relatively better performance at predicted probabilities <0.4 (Fig 5B). However, 8 nonprogressors were classified as high risk (score ≥8; ≥70% risk), possibly due to overfitting during data-driven threshold derivation, which may limit its precision in identifying high-risk progressors. These findings align with prior validation studies of Leiden CPR in individuals with UA [64]. Differences between the derivation cohort (UA) and validation cohort (without clinical synovitis) likely contributed to the observed performance, highlighting a limitation of the validation set. In contrast, most RPRs revealed a less favourable balance of predictive accuracy, calibration and discrimination, reflecting variability in populations, outcomes, or areas for improvement during model development. Simplifying a prediction model by omitting clinically meaningful predictors may reduce its performance [65], whereas overly complex models or those requiring biomarker measurements may be difficult to apply in clinical practice.

An important consideration for clinical utility is how risk thresholds were determined. Many RPRs used data-driven thresholds, such as maximising sensitivity and specificity, which may not reflect meaningful clinical decisions. Decision-analytic approaches, like decision curve analysis, evaluate trade-offs between true and false positives across a range of risk thresholds. In this study, we did not perform formal net-benefit calculations. Given the relatively small number of high-risk individuals (95% with ACPA ≥3× upper limit normal) and poor to modest discrimination in validated RPRs (maximum C-statistic 0.65), such estimates would likely be uncertain. Future work should prioritise threshold selection in the context of clinical consequences to better link predictive performance and practical utility. Further evaluation of additional predictors, including biomarkers or advanced imaging findings, may also help refine future risk prediction models for RA.

Our study has several limitations. The sample size for external validation was smaller than recommended [66,67]^,^ reducing confidence in generalizability. In addition, some predictors (eg, RF, anti-CCP, ESR, and CRP) were measured in local laboratories with different reference ranges, which may affect comparability and model performance. A key consideration is that validation cohort represents a higher-risk population than many other cohorts without clinical synovitis, meaning only a subset of individuals would be of comparable risk. This difference in baseline risk, together with heterogeneity in outcome definitions and timing and bias introduced by missing data, may have influenced the observed validation performance metrics. Nevertheless, this cohort remains clinically relevant, as it reflects the population in which such RPRs are likely to be applied in practice, particularly when considering targeted interventions such as biological therapies. Finally, many validation studies were reported separately, which may overestimate the RoB due to lack of external validation. Thus, the results should be interpreted cautiously, and RRPs applied judiciously, bearing in mind transportability: a tool that performs well in 1 at-risk cohort at a specific point in time may not necessarily perform well in similar but slightly different cohorts at different points in time.

Overall, our validation findings highlighted that relying on a single performance metric, such as discrimination, provides an incomplete assessment of clinical usefulness. Discrimination in the validation cohort was consistently lower than in the derivation cohorts, as observed previously [[67], [68]−69], likely reflecting overfitting or population heterogeneity [70]. Although good calibration is desirable, even rules with imperfect calibration may retain clinical utility [34]. Adherence to reporting standards, such as transparent reporting of a multivariable prediction model for individual prognosis or diagnosis [71] or PROBAST [27], together with robust statistical methods, is essential to improve the development, validation, and clinical applicability of RA RPRs. Existing RA RPRs demonstrated inconsistent performance when externally validated, highlighting the need for transparent reporting, robust model development, and independent validation before clinical implementation. Their role in routine clinical practice remains uncertain.

## CRediT authorship contribution statement

**Marianna Jasenecova:** Writing – review & editing, Writing – original draft, Visualization, Software, Methodology, Formal analysis, Conceptualization. **Gabrielle Harker:** Writing – review & editing, Validation. **Maryam Adas:** Writing – review & editing, Methodology. **Sumera Qureshi:** Writing – review & editing, Validation. **Laurence Duquenne:** Writing – review & editing, Resources. **Michelle Wilson:** Writing – review & editing, Validation, Resources. **Elizabeth M A Hensor:** Writing – review & editing, Validation, Resources. **Paul Emery:** Writing – review & editing, Resources. **Kulveer Mankia:** Writing – review & editing, Resources. **Katie Bechman:** Writing – review & editing, Conceptualization. **Kathryn Steel:** Writing – review & editing, Resources. **Yang Luo:** Writing – review & editing, Resources. **Sam Norton:** Writing – review & editing, Supervision, Methodology, Conceptualization. **Andrew P Cope:** Writing – review & editing, Visualization, Supervision, Methodology, Conceptualization.

## Competing interests

MJ reports a relationship with Bristol Myers Squibb that includes: funding grants. APC reports a relationship with Bristol Myers Squibb that includes: funding grants. PE reports a relationship with Bristol Myers Squibb that includes: funding grants. KM reports a relationship with Bristol Myers Squibb that includes: funding grants. The other authors have nothing to declare.

## Appendix Supplementary materials

Image, application 1

## Footnotes

Supplementary material associated with this article can be found in the online version at doi:10.1016/j.ero.2026.100228.
