# Supplementary material for: Risk prediction rules in individuals at risk of rheumatoid arthritis: systematic review and validation using the APIPPRA cohort
Source: EULAR Rheumatol Open. 2026 Aug 24;2(3):100228. doi: 10.1016/j.ero.2026.100228 (PMC13527503; doi:10.1016/j.ero.2026.100228)
Supplement: Supplementary file 1 [file mmc1.docx]

Contents

[Figure S1. Search terms used in the systematic review	2](#_Toc221888224)

[Figure S2. Key summary characteristics of the studies included in the systematic review	4](#_Toc221888225)

[Table S1. Summary of characteristics of the studies included in the systematic review	5](#_Toc221888226)

[Table S2. Summary of RA risk prediction rules characteristics	15](#_Toc221888227)

[Figure S3. Summary of Risk of bias and Applicability assessment	23](#_Toc221888228)

[Table S3. Missing data summaries	24](#_Toc221888229)

[A.	Van der Helm-van Mil, 2008, year 1 outcome	24](#_Toc221888230)

[B.	Ha, 2012, year 1 outcome	24](#_Toc221888231)

[C.	Rakieh, 2015, PD model, year 2 outcome	24](#_Toc221888232)

[D.	Rakieh, 2015, SE model, year 2 outcome	24](#_Toc221888233)

[E.	Rakieh, 2015, PD+SE model, year 2 outcome	24](#_Toc221888234)

[F.	Rakieh, 2015, nonPD/nonSE model, year 2 outcome	24](#_Toc221888235)

[G.	Ghosh, 2016, year 1 outcome	25](#_Toc221888236)

[H.	Di Mateo, 2020, year 1 and year 3 outcome	25](#_Toc221888237)

[I.	Van Boheemen, 2023, combined group, year 2 outcome	25](#_Toc221888238)

[J.	Van Boheemen, 2023, seropositive subgroup, year 2 outcome	25](#_Toc221888239)

[K.	Duquenne, 2023, simple score, year 1 outcome	26](#_Toc221888240)

[L.	Duquenne, 2023, comprehensive categorical score, year 1 outcome	26](#_Toc221888241)

[M.	Duquenne, 2023, comprehensive categorical score, year 5 outcome	26](#_Toc221888242)

[N.	Duquenne, 2023, comprehensive continuous score, year 1 outcome	26](#_Toc221888243)

[O.	Duquenne, 2023, comprehensive continuous score, year 5 outcome	27](#_Toc221888244)

[Figure S4. Distribution of prediction scores and calibration evaluation in the independent APIPPRA placebo validation cohort	28](#_Toc221888245)

[A.	Van der Helm-van Mil, 2008, year 1 outcome	28](#_Toc221888246)

[B.	Ha, 2012, Simplified prediction model, year 1 outcome	29](#_Toc221888247)

[C.	Rakieh, 2015, nonPD/nonSE model, year 2 outcome	30](#_Toc221888248)

[D.	Rakieh, 2015, PD model, year 2 outcome	31](#_Toc221888249)

[E.	Rakieh, 2015, SE model, year 2 outcome	32](#_Toc221888250)

[F.	Rakieh, 2015, PD+SE model, year 2 outcome	33](#_Toc221888251)

[G.	Ghosh, 2016, year 1 outcome	34](#_Toc221888252)

[H.	Di Mateo, 2020, year 1 outcome	35](#_Toc221888253)

[I.	Di Mateo, 2020, year 3 outcome	36](#_Toc221888254)

[J.	Van Boheemen, 2023, combined group, year 2 outcome	37](#_Toc221888255)

[K.	Van Boheemen, 2023, seropositive subgroup, year 2 outcome	38](#_Toc221888256)

[L.	Duquenne, 2023, simple score, year 1 outcome	39](#_Toc221888257)

[M.	Duquenne, 2023, comprehensive categorical score, year 1 outcome	40](#_Toc221888258)

[N.	Duquenne, 2023, comprehensive categorical score, year 5 outcome	41](#_Toc221888259)

[O.	Duquenne, 2023, comprehensive continuous score, year 1 outcome	42](#_Toc221888260)

[P.	Duquenne, 2023, comprehensive continuous score, year 5 outcome	43](#_Toc221888261)

Figure S1. Search terms used in the systematic review

Discrimination, overall predictive accuracy and calibration assessment

Discrimination was assessed by the area under the receiver operating characteristic curve (AUROC), which usually ranges between 0.5 (no discrimination) to 1 (perfect discrimination), with higher values indicating better discrimination^1^. Although some models were originally developed using time-to-event outcomes, predicted risks were reported at fixed time horizons; therefore, outcomes were evaluated as binary events at the corresponding time points, allowing all models to be assessed using the same outcome definition. For binary outcomes, the AUC is equivalent to the C-statistic, representing the probability that for a random selected pair of individuals with different outcomes, the individual with an outcome has a higher predicted risk than the individual without an outcome^2,3^. In this study, AUC was classified as poor (C-statistic 0.5-0.6), moderate (0.6-0.7), or fair (0.7-0.8). Overall RPR accuracy was assessed using the Brier score, which ranges from 0 for perfect accuracy to 1 for imperfect inaccuracy^4^, with values <0.25 indicating more accurate prediction overall^5^. Calibration, reflecting how closely predicted probabilities match observed event rates, was evaluated using calibration plots and the observed-to-expected (O/E) event ratio, with O/E>1 indicating overprediction and O/E<1 indicating underprediction. Ideal calibration was indicated by a calibration intercept of 0, a calibration slope of 1, and O/E ratio of 1. The 95% CIs for the O/E ratios were calculated using a binomial test for the number of observed events. Negative intercept values indicated that the model overpredicts risks on average and positive intercept values indicate underprediction. A calibration slope <1 indicated that the predictions are overly extreme (high risks too high, low risks too low), while slopes >1 indicate overly conservative predictions^6^.

Figure S2. Key summary characteristics of the studies included in the systematic review

Table S1. Summary of characteristics of the studies included in the systematic review

| **Undifferentiated/Inflammatory/Suspected RA** | | | | | | | | | | | | |
| --- | --- | --- | --- | --- | --- | --- | --- | --- | --- | --- | --- | --- |
| **First Author, Year** | **Study region** | | **Study setting** | **Study design** | **Clinical Inclusion criteria** | **Exclusion criteria** | **Outcome** | **Enrolment period** | **No of RPRs/models** | **RPR/model** | **Outcome Type and Time occurrence** | **Follow-up period** |
| Suenaga, 1998 | Japan | Hospital outpatient department | | Prospective cohort | Patients with suspected RA* (2 out of 5 modified 1958 criteria for possible RA: morning stiffness, tenderness or pain on motion with a history of recurrence or persistence for 3 weeks, history or observation of joint swelling, subcutaneous nodules) observed by a physician, raised ESR or CRP | Absent bone and joint changes on X-rays | RA (Fulfilment of 1987 ARA revised criteria for RA; Early RA with disease duration of <1 year without bony changes and established RA with disease duration >1 year and bony changes.) | Beginning 1990-December 1991 | 1 | Model with serum interleukin-2 receptor | Binary, 1 year | 1-5 years |
| Van der Helm-van Mil, 2007 | Netherlands | Leiden EAC | | Prospective cohort | Patients with recent-onset UA |  | RA (Fulfilment of ACR 1987 criteria for RA) | From 1993 | 1 | Leiden CPR | Binary, 1 year | min 1 year, 8 ± 3 years (mean ± sd) |
| Van der Helm-van Mil, 2008 | Netherlands | Leiden EAC | | Prospective cohort | Patients with recent-onset UA |  | RA (Fulfilment of ACR 1987 criteria for RA) | From 1993 | 1 | Rederived Leiden CPR | Binary, 1 year | min 1 year, 8 ± 3 years (mean ± sd) |
| Tamai, 2009 | Japan | EAC at Nagasaki University | | Prospective cohort | Patients with UA |  | RA (Fulfilment of ACR 1987 criteria for RA) | From 2001 | 1 | Prediction rule by serologic variables and MRI | Binary, 1 year | min 1 year |
| Salaffi, 2010 | Italy | EAC at Università Politecnica delle Marche, Ancona | | Prospective cohort | Patients with recent-onset UA | DMARDs or steroid treatment | RA (Fulfilment of ACR 1987 criteria for RA) | No information | 1 | Prediction rule by clinical + power Doppler US | Binary, 1 year | mean 12 months (range 11-14 months) |
| Duer-Jensen, 2011 | Denmark | EAC at Herlev, Hvidovre, Odense Hospitals, King Christian Xth Hospital in Gråsten, through 4 rheumatologists in private practice | | Nested case–control within prospective cohort | Patients with early UA (With ≥2 tender joints and/or ≥2 swollen joints for >6 weeks but <24 months, symptom duration between 6 weeks and 24 months, and no contraindications for MRI. Healthy subjects with no joint symptoms or skin manifestations. | Patients with verification of a specific rheumatic disease after routine clinical, biochemical, and radiographic examinations at baseline | RA (Fulfilment of ACR 1987 criteria for RA) | June 2006-July 2008 | 1 | Prediction model by clinical, serological, and MRI | Binary | >= 12 months (median 17 months, range 12–23 months) |
| Ha, 2012 | Korea | Yonsei University Medical Center | | Retrospective cohort | Patients with UA defined as IA that does not fulfil classification criteria for rheumatic diseases |  | RA (Fulfilment of ACR 1987 criteria for RA) | January 2004-December 2007 | 1 | Simplified prediction model | Binary | min 6 months, mean 17.4 months |
| Ghosh, 2016 | Kolkata, India | Nilratan Sircar Medical College and Hospital | | Prospective cohort | Patients with IP with history <2 years duration, not fulfilling diagnostic criteria for any rheumatic diseases / UA | Current or previous DMARDs use | RA (Fulfilment of ACR 1987 criteria for RA) | March 2011-March 2012 | 1 | Simplified prediction formula using Leiden CPR | Binary, 1 year | 12 months |
| Fujii, 2025 | Japan | Hospitals | | Retrospective cohort | Patients with seronegative UA:  KURAMA cohort – training dataset  ANSWER – validation dataset | (i) Less than 18 years old  (ii) history of malignancy, (iii) known autoimmune disease or treated with immunosuppressants, (iv) <6 months follow up, (v) RF and/or ACPA positive, (vi) previous RA diagnosis, (vii) met ACR/EULAR 2010 RA classification at baseline | RA ((i) diagnosis from medical records, ii) MRI/US synovitis, tenosynovitis, bone oedema and bone erosions, iii) 2010 ACR/EULAR classification criteria for RA, iv) radiographic progression) | KURAMA (2011 – 2022), ANSWER (from 2018) | 1 | FNN model using SHAP | Binary,  37 days [28–76 days]  Mean [Q1-Q3] | Minimum 6 months |
| Salehi, 2025 | Germany | Erlangen University Hospital | | Prospective cohort | Individuals with joint pain or related symptoms and positive for anti-CCP antibodies |  | RA (Fulfilment of 2010 ACR/EULAR RA classification criteria) | 09/04/2010-04/10/2023 | 1 | RSF model using SHAP | Time to outcome | mean 1.4 years for RA progressors and 7.06 years for non-progressors |

| **Individuals at risk of RA** | | | | | | | | | | | |
| --- | --- | --- | --- | --- | --- | --- | --- | --- | --- | --- | --- |
| **First Author, Year** | **Study region** | **Study setting** | **Study design** | **Clinical Inclusion criteria** | **Exclusion criteria** | **Outcome** | **Enrolment period** | **No of RPRs/models** | **RPR/model** | **Outcome Type and Time occurrence** | **Follow-up period** |
| Van de Stadt, 2013 | Netherlands | Rheumatology outpatient clinics in Amsterdam area | Prospective cohort | aCCP and/or IgM-RF positive individuals with (a history of) arthralgia | Individuals with arthritis at baseline, a negative aCCP and IgM-RF status on second analysis, age >70 years, previous DMARD or recent glucocorticoid treatment, without follow-up | Arthritis (Fulfilment of ACR 1987 and 2010 ACR/EULAR RA criteria) | August 2004-June 2011 | 1 | Simplified prediction rule | Time to outcome | 32 months (IQR: 13–48 months) |
| Pratt, 2013 | UK | Newcastle EAC | Prospective cohort | Individuals ≥16 years old with inflammatory new-onset arthralgia | Individuals with clinically swollen joints** | PIA (Fulfilment of ACR 1987 criteria for RA, psoriatic arthritis, enteropathic arthritis, ankylosing spondylitis, undifferentiated spondyloarthropathy, connective tissue disease, other IAs and self-limiting inflammatory/reactive arthritis) | September 2006-November 2009 | 2 | Risk metric calculation tool with and without MSUS | Binary | ≥12 months (median 28 months) |
| Rakieh, 2015 | UK | Primary care services in Yorkshire and rheumatology EAC in Leeds | Prospective cohort | Individuals >18 years old with new onset MSK symptoms, anti-CCP positive | History of IA diagnosed by a rheumatologist; presence of clinically detected IA at baseline confirmed by a rheumatologist; use of DMARDs. | IA (At least one tender and swollen joint confirmed by a rheumatologist) | No information | 4 | PD, SE, PD+SE, non-PD/non-SE | Time to outcome, 1, 2 years and end of follow up | 19.8 months  (0.1-69.0)  median (range) |
| Van Steenbergen, 2017 | Netherlands, UK, Romania, Hungary, Germany, Portugal, Spain, France, Czech Republic, Sweden, Austria, Poland | Outpatient clinics | Consensus-based / Delphi process | CSA Cohort: Individuals with arthralgia considered at increased risk of RA based on history taking and physical examination  no-CSA Cohort: no diagnosis or explanation for arthralgia but judged not be at risk of RA | Patients with evident diagnoses, such as fibromyalgia or osteoarthritis at presentation; clinically detectable arthritis | CSA for progression to RA | No information | 1 | Classification criteria for CSA | Binary | No information |
| Erlandsson, 2018 | Sweden | Rheumatology Clinic at the Sahlgrenska University Hospital of Gothenburg | Prospective cohort | Individuals with unexplained arthralgia | Individuals < 18 years old, registered deceased at follow up, patients with known diagnosis of inflammatory joint or spine disease, systemic rheumatic disease, gout and generalized pain conditions. | Development of polyarthritis (Fulfilment of 2010 ACR/EULAR Criteria for RA) | 5 November 2012-4 November 2013 | 2 | Model A: clinical, serological and laboratory parameters incl. age, Model B: clinical, serological and laboratory parameters incl. CRP | Time to outcome | 48 months |
| Matthijssen, 2019 | Netherlands | Leiden rheumatology outpatient clinic | Prospective cohort | CSA individuals with recent-onset (<12 months) arthralgia affecting hands or feet and considered at risk for progression to RA as judged by rheumatologist | Patients presented with clinical arthritis or if another explanation for the symptoms (e.g. osteoarthritis, fibromyalgia). | Main outcome: development of clinically apparent IA (≥ 1 tender and swollen joint confirmed by a rheumatologist)  Secondary outcome: development of RA defined as clinical diagnosis and fulfilment of the 1987 or the 2010 classification criteria for RA.) | April 2012-April 2015 | 1 | Online risk score using MRI findings of number of joints with subclinical inflammation, presence of MCP extensor peritendinitis | Time to outcome | 12 months |
| Di Matteo, 2020 | UK | Research clinic in Leeds | Prospective cohort | Anti-CCP positive at-risk individuals with MSK symptoms | Individuals with clinical synovitis | IA: clinical synovitis in ≥1 joint.  RA: fulfilment of 2010 ACR/EULAR RA classification criteria) | June 2008-December 2019 | 2 | Model A with clinical, serological and ultrasound parameters at 1 year outcome and model B at 3 years outcome | Time to outcome, 1 year and 3 years | 1 year |
| Van Boheemen, 2023 | Netherlands, UK, Sweden, and Switzerland | Reade, Amsterdam; Sandwell and West Birmingham and the University Hospitals Birmingham; Karolinska University Hospital; and the University Hospital of Geneva | Combined prospective cohorts | Individuals at risk of developing RA: ACPA and/or RF positive, or the presence of relevant symptoms (e.g. arthralgia suspicious for progression to RA based on clinical expertise) with or without RA- specific antibodies | Clinically apparent arthritis | Clinical arthritis (≥1 swollen joint, as observed by a rheumatologist) | March 2014-November 2017 | 2 | RPR for Combined group and seropositive subgroup | Time to outcome, 24 months | 24 months (17 - 36)  median, (IQR) |
| Duquenne, 2023 | UK | Primary care centers and rheumatology secondary care clinics in Leeds | Prospective cohort | Individuals with new MSK symptoms, ACPA positive | A history of DMARDs exposure or clinical synovitis | IA: defined by the presence of clinical synovitis | June 2008-November 2021 | 4 | Simple, Comprehensive categorical, Comprehensive continuous, Exploratory score | Time to outcome, 1 and 5 years | Median 233 weeks, min 48 weeks until IA occurred, recruitment for 13 years |
| Prajzlerova, 2024 | Czech Republic | Outpatient clinic of the Institute of Rheumatology | Prospective cohort | ARRA cohort: (i) ACPA (anti-CCP and/or anti-MCV) positive and arthralgia or (ii) CSA (2017 EULAR criteria) irrespective of ACPA | Evidence of arthritis on the examination | Clinical arthritis (≥1 swollen joint observed by a trained rheumatologist) | No information | 2 | Logistic regression model for all and for ACPA+ subgroup | Binary, 1 year | 18 (6.4–32.5) months, median (IQR) |
| Frazzei, 2025 | Netherlands | Rheumatology outpatient clinic in Reade, Amsterdam | Prospective cohort | Individuals with arthralgia, ACPA and/or IgM-RF positive | Presence of arthritis at physical examination, glucocorticoids in the past 3 months, and had DMARDs | Arthritis (≥1 swollen joint, independently evaluated by 2 rheumatologists) | 2004-2019 | 1 | Absolute risk by clinical variables | Time to outcome, 1,3 and 5 years | Non-progressors median [IQR]: 58 [25-61]) months |
| Van Steenbergen, 2025 | Netherlands, UK, Germany, Italy, Austria | Secondary care clinics in Amsterdam, Leiden, Rotterdam, TREAT EARLIER multicenters, Birmingham, Leeds, Erlangen, Rome, and Vienna | Combined prospective cohorts | Individuals at risk for RA because of autoantibody positivity with any arthralgia/MSK symptoms; or with arthralgia based on the rheumatologists’ expert opinion | DMARD treatment or clinically apparent IA at inclusion | IA and secondary RA (IA ascertained by consensus; Fulfilment of 2010 ACR/EULAR RA classification criteria) | No information | 5 | 3 extended and 2 simplified risk stratification criteria | Binary, 1 year | 12 months |

| **Unknown clinical status** | | | | | | | | | | | |
| --- | --- | --- | --- | --- | --- | --- | --- | --- | --- | --- | --- |
| **First Author, Year** | **Study region** | **Study setting** | **Study design** | **Clinical Inclusion criteria** | **Exclusion criteria** | **Outcome** | **Enrolment period** | **No of RPRs/models** | **RPR/model** | **Outcome Type and Time occurrence** | **Follow-up period** |
| Deane, 2010 | Maryland, USA | WRAMC Rheumatology Clinic & DoDSR | Nested case–control within retrospective cohort | Individuals with seropositive RA with samples from minimum 6 months pre-RA diagnosis, matching controls without RA | Seronegative RA | RA (Fulfilment of >=4 of ACR 1987 revised criteria for RA) | 1989-2003 | 1 | Model with age and cytokine/chemokine counts | Time to outcome | 6.6 ± 3.7 years, mean ± SD, range 0.06–13.67 years |
| Lahiri, 2014 | UK | EPIC, Norfolk linkage with the Norfolk Arthritis Register | Prospective cohorts combined with registry linkage | EPIC-Norfolk Study: Individuals aged 40–79 years, who completed baseline questionnaire, clinical examination and provided a blood sample, who were alive at the time of 2010 through linkage with UK Office of National Statistics. | No alternative diagnosis subsequently given by rheumatologist and symptom onset before 2010 | IP defined as inflammation of ≥2 peripheral joints persisting for at least 4 weeks, onset after 1989, confirmed by research nurse. Secondary outcome: RA (Fulfilment of ACR 1987 criteria for RA) and seropositive IP. | 1993-1997 | 2 | Model 1 for men and model 2 for women | Time to outcome | median (IQR) of 14.2 (12.9, 15.3) years |
| Zaim, 2025 | Maryland, USA | DoDSR, The Armed Forces Health Longitudinal Technology Application | Nested case–control within retrospective cohort | ACPA positive individuals with RA and pre/post-RA blood samples and matched controls without RA |  | RA (Fulfilment of 1987 ACR classification criteria for RA or after clinical diagnosis) or diagnosed by board-certified rheumatologist | 1991 - 2012 | 1 | Biomarker RA onset classifier | Time to outcome, 3 years | >20 years timespan of samples |

Quality checks of the extracted information were performed independently by S.Q.

*41/47 individuals presented no swollen joints at first visit

** SJC≥1 observed in 46% individuals at baseline, 0 (0 to 3) median (IQR)

ACPA=Anti-Citrullinated Protein Antibodies; ACR=American College of Rheumatology; Anti-CCP=Anti-Cyclic Citrullinated Peptide; CPR=Clinical Prediction Rule; CRP=C-Reactive Protein; CSA=Clinically Suspect Arthralgia; DMARD=Disease-Modifying Antirheumatic Drug; DoDSR=Department of Defense Serum Repository; EAC=Early Arthritis Clinic; ESR=Erythrocyte Sedimentation Rate; EPIC=The European Prospective Investigation of Cancer; EULAR=European Alliance of Associations for Rheumatology; FNN=Feedforward Neural Network; IA=Inflammatory Arthritis; IgM-RF=Immunoglobulin M Rheumatoid Factor; IP=Inflammatory polyarthritis; IQR=Interquartile range; Anti-MCV=Antibodies against Modified Citrullinated Vimentin; MSK=musculoskeletal; PD=Power Doppler; PIA=Persistent Inflammatory Arthritis; RA=Rheumatoid Arthritis; RF=Rheumatoid Factor; RPR=Risk Prediction Rule; SE=Shared Epitope; RSF=Random Survival Forest; SHAP=SHapley Additive exPlanations; UA=Undifferentiated Arthritis; WRAMC=Walter Reed Army Medical Center

Table S2. Summary of RA risk prediction rules characteristics

| **First Author, Year** | **Risk prediction rule/model** | **Modelling method** | **Risk Prediction rule format** | **Other formats** | **Sample size** | **Events n (%)** | **Approx. no. Candidate predictors** | **No Final predictors (parameters):** | **Clinical*** | **Serology** | **Imaging** | **Laboratory** | **EPV/EPP** | **Selection of candidate predictors** | **Selection of final predictors** | **Validation**** | **Performance measures** |
| --- | --- | --- | --- | --- | --- | --- | --- | --- | --- | --- | --- | --- | --- | --- | --- | --- | --- |
| Suenaga, 1998 | Model with serum interleukin-2 receptor | Logistic regress. | Regress. formula |  | 47 | 22 (46.8) | 8 | 3 | x | x | NA | x | <10 | Univariable associations | p-value | Int: N Ext: N | Cal: Not evaluated Dis: Sensitivity, Specificity Ov: R-squared |
| Van der Helm-van Mil, 2007 | Leiden CPR | Logistic regress. | Point scoring system |  | 570 | 177 (31.1) | 20 | 9 (16) | x | x | NA | NA | <10 | All available predictors | Backward elimination | Int: Y Ext: Y | Cal: Not evaluated Dis: AUC graph / AUC Ov: R-squared |
| Van der Helm-van Mil, 2008 | Rederived Leiden CPR | Logistic regress. | Point scoring system |  | 570 | 177 (31.1) | 20 | 9 (16) | x | x | NA | NA | <10 | All available predictors | Backward elimination | Int: Y Ext: Y | Cal: Not evaluated Dis: AUC graph / AUC Ov: Not evaluated |
| Tamai, 2009 | Prediction rule by serologic variables and MRI | Logistic regress. | Point scoring system | Regress. coefficients | 129 | 75 (58.1) | 7 | 3 | NA | x | x | NA | <10 | Unclear | Pre-specified model (not selection) | Int: N Ext: N | Cal: Not evaluated Dis: Sensitivity, specificity, PPV and NPV Ov: Not evaluated |
| Salaffi, 2010 | Prediction rule by clinical + power Doppler US | Logistic regress. | Point scoring system |  | 149 | 62 (41.6) | 12 | 5 (8) | x | x | x | NA | <10 | Univariable associations | Backward elimination | Int: N Ext: N | Cal: Not evaluated Dis: C-Statistic / AUC graph  Ov: Not evaluated |
| Duer-Jensen, 2011 | Prediction model by clinical, serological, and MRI | Logistic regress. | Regress. formula |  | 140 (116 pts. and 24 HC) | 27 (19.3) | 20 | 4 | x | x | x | NA | <10 | Univariable associations | Backward elimination | Int: N Ext: N | Cal: Not evaluated Dis: Sensitivity, Specificity, PPV, NPV Ov: R-squared |
| Ha, 2012 | Simplified prediction model | Logistic regress. | Point scoring system |  | 164 | 32 (19.5) | 18 | 7 | x | x | x | NA | <10 | Univariable associations | Backward elimination | Int: N Ext: N | Cal: Not evaluated Dis: C-Statistic / AUC graph  Ov: Not evaluated |
| Ghosh, 2016 | Simplified prediction formula using Leiden CPR | Logistic regress. | Regress. formula |  | 50 | 27 (54.0) | 11 | 3 | x | NA | NA | NA | <10 | Univariable associations | Pre-specified model (not selection) | Int: N Ext: N | Cal: Not evaluated Dis: C-Statistic / AUC graph  Ov: Not evaluated |
| Fujii, 2025 | FNN model using SHAP | Machine learning FNN | SHAP values |  | 210 | 57 (27.1) | 16 | 16 | x | x | NA | x | <10 | Based on prior knowledge | Pre-specified model (not selection) | Int: Y Ext: Y | Cal: Not evaluated Dis: C-Statistic / AUC graph / Sensitivity and Specificity Ov: Not evaluated |
| Van de Stadt, 2013 | Prediction rule for the development of arthritis in seropositive arthralgia patient | Cox regress. | Point scoring system |  | 374 | 131 (35.0) | 18 | 9 (11) | x | x | NA | NA | <10 | Univariable associations | Backward elimination | Int: Y Ext: N | Cal: Not evaluated Dis: C-Statistic / AUC graph / Risk group curves  Ov: R-squared |
| Pratt, 2013 | Risk metric calculation tool without MSUS | Logistic regress. | Point scoring system | Regress. coefficients | 379 | 162 (42.7) | 12 | 7 (9) | x | x | x | NA | 10 to 20 | Univariable associations | Backward elimination | Int: Y Ext: N | Cal: Not evaluated Dis: C-Statistic / AUC graph  Ov: R-squared |
| Pratt, 2013 | Risk metric calculation tool with MSUS | Logistic regress. | Point scoring system | Regress. coefficients | 379 | 162 (42.7) | 14 | 10 (12+) | x | x | NA | NA | 10 to 20 | Univariable associations | Backward elimination | Int: Y Ext: N | Cal: Not evaluated Dis: C-Statistic / AUC graph  Ov: R-squared |
| Rakieh, 2015 | PD model | Cox regress. | Point scoring system | Regress. coefficients | 100 | 50 (50.0) | 18 | 4 | x | x | x | NA | <10 | Univariable associations | HR ≥1.5 | Int: N Ext: N | Cal: Not evaluated Dis: C-Statistic / Risk group curves  Ov: Not evaluated |
| Rakieh, 2015 | SE model | Cox regress. | Point scoring system | Regress. coefficients | 92 | 50 (50.0) | 18 | 4 | x | x | NA | x | <10 | Univariable associations | HR ≥1.5 | Int: N Ext: N | Cal: Not evaluated Dis: C-Statistic / Risk group curves  Ov: Not evaluated |
| Rakieh, 2015 | PD+SE model | Cox regress. | Point scoring system | Regress. coefficients | 92 | 50 (50.0) | 18 | 5 | x | x | x | x | <10 | Univariable associations | HR ≥1.5 | Int: N Ext: N | Cal: Not evaluated Dis: C-Statistic / Risk group curves  Ov: Not evaluated |
| Rakieh, 2015 | Non-PD/non-SE model | Cox regress. | Point scoring system | Regress. coefficients | 100 | 50 (50.0) | 18 | 3 | x | x | NA | NA | <10 | Univariable associations | HR ≥1.5 | Int: N Ext: N | Cal: Not evaluated Dis: C-Statistic / Risk group curves  Ov: Not evaluated |
| Erlandsson, 2018 | Model A with clinical and laboratory | Cox regress. | Point scoring system |  | 180 | 32 (17.8) | 17 | 6 | x | x | NA | x | <10 | Univariable associations | Forward selection | Int: N Ext: N | Cal: Not evaluated Dis: C-Statistic / AUC graph / Log-rank test / Risk group curves  Ov: Not evaluated |
| Erlandsson, 2018 | Model B with clinical, laboratory, and demographics | Cox regress. | Point scoring system |  | 180 | 32 (17.8) | 17 | 6 | x | x | NA | x | <10 | Univariable associations | Forward selection | Int: N Ext: N | Cal: Not evaluated Dis: C-Statistic / AUC graph / Log-rank test / Risk group curves  Ov: Not evaluated |
| Matthijssen, 2019 | Online risk score using MRI | Cox regress. | Regress. coefficients |  | 225 | 34 (15.1) | 14 | 2 (3) | NA | NA | x | NA | <10 | Univariable associations | Backward elimination | Int: Y Ext: Y | Cal: Calibration plot / Slope / HL test  Dis: C-Statistic / AUC graph / Risk group curves  Ov: Not evaluated |
| Di Matteo , 2020 | Model A for 1 year outcome | Cox regress. | Regress. coefficients |  | 419 | 123/400 (30.7%) IA, 95/123 (77.2%) RA | 9 | 4 | x | x | x | NA | 10 to 20 | Univariable associations | Backward elimination | Int: N Ext: N | Cal: Not evaluated Dis: Not evaluated Ov: R-squared |
| Di Matteo , 2020 | Model B for 3 year outcome | Cox regress. | Regress. coefficients |  | 419 | 123/400 (30.7%) IA, 95/123 (77.2%) RA | 9 | 4 | x | x | x | NA | 10 to 20 | Univariable associations | Backward elimination | Int: N Ext: N | Cal: Not evaluated Dis: Not evaluated Ov: R-squared |
| Van Boheemen, 2023 | Combined group | Cox regress. | Point scoring system |  | 214 | 58 (27.1) | 69 | 4 | x | NA | NA | NA | <10 | Univariable associations | Forward selection | Int: Y Ext: N | Cal: Not evaluated Dis: C-Statistic / AUC value & 95% CI Ov: Not evaluated |
| Van Boheemen, 2023 | Seropositive subgroup | Cox regress. | Point scoring system |  | 214 | 58 (27.1) | 69 | 3 (4) | x | NA | NA | NA | <10 | Univariable associations | Forward selection | Int: Y Ext: N | Cal: Not evaluated Dis: C-Statistic / AUC value & 95% CI Ov: Not evaluated |
| Duquenne, 2023 | Simple score | Logistic regress. | Point scoring system |  | 455 | 148 (32.5) | 15 | 4 (5) | x | x | NA | NA | <10 | Univariable associations | Backward elimination | Int: Y Ext: N | Cal: Calibration plot / Slope  Dis: C-Statistic / AUC graph  Ov: Not evaluated |
| Duquenne, 2023 | Comprehensive categorical | Cox regress. | Point scoring system |  | 455 | 70 (15.4%) at 1 year and 148 (32.5%) at 5 years | 20 | 12 (13) | x | x | x | x | <10 at 1 year and 5 years | All available predictors | LASSO selection | Int: Y Ext: N | Cal: Calibration plot / Slope  Dis: C-Statistic / AUC graph / Gönen and Hellers K-statistic Ov: Not evaluated |
| Duquenne, 2023 | Comprehensive continuous | Cox regress. | Regress. coefficients | Calculator | 455 | 70 (15.4%) at 1 year and 148 (32.5%) at 5 years | 19 | 14 | x | x | x | x | <10 at 1 year and 5 years | All available predictors | LASSO selection | Int: Y Ext: N | Cal: Calibration plot / Slope  Dis: C-Statistic / AUC graph / Gönen and Hellers K-statistic Ov: Not evaluated |
| Duquenne, 2023 | Exploratory score | Cox regress. | Regress. coefficients | Calculator | 455 | 70 (15.4%) at 1 year and 148 (32.5%) at 5 years | 22 | 16 | x | x | x | x | <10 at 1 year and 5 years | All available predictors | LASSO selection | Int: Y Ext: N | Cal: Calibration plot / Slope  Dis: C-Statistic / AUC graph / Gönen and Hellers K-statistic Ov: Not evaluated |
| Van Steenbergen, 2017 | EULAR defined CSA | Logistic regress. | Point scoring system | Regress. Coefficients, weighted score | 139 | No information | 16 | 7 | x | NA | NA | NA | No information | Modified Delphi procedure & univariable analyses | p-value | Int: N Ext: N | Cal: No information Dis: C-Statistic Ov: No information |
| Frazzei, 2025 | Absolute risk by clinical variables | Cox regress. | Point scoring system |  | 617 | 208 (33.7) | 12 | 6 | x | x | NA | NA | 10 to 20 | Based on prior knowledge/  literature | p-value | Int: Y Ext: N | Cal: Not evaluated Dis: Sensitivity, Specificity, PPV, NPV Ov: Not evaluated |
| Van Steenbergen, 2025 | Extended clinical and serological | Logistic regress. | Point scoring system |  | 728 | 134 (18.4) | 31 | 7 | x | x | NA | NA | <10 | Univariable associations | LASSO selection | Int: Y Ext: N | Cal: Calibration plot / Slope / CITL  Dis: C-Statistic / Sensitivity, Specificity Ov: Not evaluated |
| Van Steenbergen, 2025 | Extended clinical and serological + US | Logistic regress. | Point scoring system |  | 835 | 153 (18.3) | 123 | 12 | x | x | x | NA | <10 | Univariable associations | LASSO selection | Int: Y Ext: N | Cal: Calibration plot / Slope / CITL  Dis: C-Statistic / Sensitivity, Specificity Ov: Not evaluated |
| Van Steenbergen, 2025 | Extended clinical and serological + MRI | Logistic regress. | Point scoring system |  | 730 | 102 (14.0) | 93 | 13 | x | x | x | NA | <10 | Univariable associations | LASSO selection | Int: Y Ext: N | Cal: Calibration plot / Slope / CITL  Dis: C-Statistic / Sensitivity, Specificity Ov: Not evaluated |
| Van Steenbergen, 2025 | Simplified clinical and serological | Logistic regress. | Point scoring system |  | 728 | 134 (18.4) | 31 | 6 (9) | x | x | NA | NA | <10 | Univariable associations | LASSO selection | Int: Y Ext: N | Cal: Calibration plot / Slope / CITL  Dis: C-Statistic / Sensitivity, Specificity Ov: Not evaluated |
| Van Steenbergen, 2025 | Simplified clinical and serological + MRI | Logistic regress. | Point scoring system |  | 730 | 102 (14.0) | 93 | 10 | x | x | x | NA | <10 | Univariable associations | LASSO selection | Int: Y Ext: N | Cal: Calibration plot / Slope / CITL  Dis: C-Statistic / Sensitivity, Specificity Ov: Not evaluated |
| Prajzlerova, 2024 | Logistic Regress. model for all | Logistic regress. | Regress. coefficients |  | 194 | 41 (21.1) | 5 | 4 | x | NA | NA | x | <10 | Based on prior knowledge | Stepwise selection | Int: N Ext: N | Cal: Not evaluated Dis: C-Statistic / AUC graph Ov: AIC |
| Prajzlerova, 2024 | Logistic Regress. model for ACPA+ subgroup | Logistic regress. | Regress. coefficients |  | 194 | 41 (21.1) | 5 | 4 | x | NA | NA | x | <10 | Based on prior knowledge | Stepwise selection | Int: N Ext: N | Cal: Not evaluated Dis: C-Statistic / AUC graph Ov: AIC |
| Salehi, 2025 | RSF model using SHAP | Machine learning techniques | SHAP values |  | 154 | 51 (33.1) | 19 | 19 | x | x | NA | NA | <10 | No information | Pre-specified model (not selection) | Int: Y Ext: N | Cal: Not evaluated Dis: C-Statistic  Ov: Not evaluated |
| Deane, 2010 | Model with age and cytokine/chemokine counts | Cox regress. | Regress. formula |  | 146 | 73 (50.0) | 24 | 2 (3+) | x | NA | NA | x | <10 | Univariable associations | p-value | Int: N Ext: N | Cal: Not evaluated Dis: Sensitivity, Specificity Ov: Not evaluated |
| Lahiri, 2014 | Model 1 for men | Cox regress. | Point scoring system | Regress. coefficients | 25,271 | 138 (0.55) | 21 | 5 (11) | x | NA | NA | NA | <10 | Univariable associations | Unclear | Int: Y Ext: N | Cal: Not evaluated Dis: C-Statistic  Ov: Not evaluated |
| Lahiri, 2014 | Model 2 for women | Cox regress. | Point scoring system | Regress. coefficients | 13,772 | 102 (0.74) | 30 | 7 (13) | x | NA | NA | NA | <10 | Univariable associations | Unclear | Int: Y Ext: N | Cal: Not evaluated Dis: C-Statistic  Ov: Not evaluated |
| Zaim, 2025 | Biomarker RA onset classifier | Logistic regress. | Regress. coefficients |  | 69 | 64 (92.8) | 204 | 8 | x | x | NA | x | <10 | All available predictors | Bootstrap and CV selection | Int: Y Ext: N | Cal: Not evaluated Dis: C-Statistic / AUC graph / Sensitivity, Specificity, and PPV  Ov: Not evaluated |

Refer to the original paper for missing data and imputation method. Quality checks of the extracted information were performed independently by S.Q. Calculation of event per variable/parameter were pre-specified in the CHARMS section with the standard formula already incorporated.

*ESR & CRP were entered as clinical predictors.

**Coded as Y=Yes or N=No/None/No information

ACPA=Anti-Citrullinated Protein Antibodies; AIC=Akaike Information Criterion; Anti-CCP=Anti-Cyclic Citrullinated Peptide; Cal=Calibration; CPR=Clinical Prediction Rule; CV=Cross-validation; CRP=C-Reactive Protein; CSA=Clinically Suspect Arthralgia; Dis=Discrimination; EPV/EPP=Event Per Variable/Parameter; ESR=Erythrocyte Sedimentation Rate; Ext=External; FDR=First-Degree Relative with RA; FNN=Feedforward Neural Network; HAQ=Health Assessment Questionnaire; HC=Healthy Controls; HR=Hazard Ratio; Int=Internal; Int=Internal; LASSO=Least Absolute Shrinkage and Selection Operator; MCP=[M](https://www.google.com/search?sca_esv=b97f4a4cdc4a391b&sxsrf=AE3TifN7tAg_SV4QFlkvC_dRfvTtUCH6Vg%3A1762190204193&q=metacarpophalangeal+joints&sa=X&ved=2ahUKEwin6paJvtaQAxUhUkEAHRrUF_kQxccNegQIOhAB&mstk=AUtExfDtFblvF_4ITWQLRA-NL90bCSu9PkkKbER4eFaA5C7G29JdhtuNG6LtOHRo6JMyIYDuvH8GfiMitkQuzXHSs_RSs2HlGE_8vCW3Sn4mwG1Av5R75OVDORf4S7q6x1yegTy3dOYDiaqQBkyO8iVTgLiIvtycme6azG22NRT-GmOkcE1nDxV6esv5XCJEILdBkcMX8jOdOyuWzOKG3piUChIiWaQxa5sXMslrvNtMo-La-11zKYkyiyV0m9H_ivXt8XHyV_knHU4AIpJLxoLWGGVi&csui=3)etacarpophalangeal; MRI=Magnetic Resonance Imaging; MSUS=Musculoskeletal Ultrasound; MTP=Metatarsophalangeal; NPV=Negative Predictive Value; Ov=Overall; PD=Power Doppler; PPV=Positive Predictive Value; RA=Rheumatoid Arthritis; Regress=Regression; RF=Rheumatoid Factor; SE=Shared Epitope; RSF=Random Survival Forest; SHAP=SHapley Additive exPlanations; SJC=Swollen Joint Count; TJC=Tender Joint Count; TSV=Tenosynovitis; US=Ultrasound; VAS=Visual Analogue Scale.

Figure S3. Summary of Risk of bias and Applicability assessment

Risk of bias and applicability were assessed for each RPR separately. An applicability assessment reflected the extent to which the main PROBAST domains: participants, predictors, and outcome, matched the review question. The proportion of RPRs rated as high, low, unclear was calculated using a pre-specified CHARMS and PROBAST template with the formula already incorporated. An unlocked version of the template was provided by authors upon request.

Table S3. Missing data summaries

A. Van der Helm-van Mil, 2008, year 1 outcome

| Final predictor | n_miss | percent |
| --- | --- | --- |
| Stiffness duration | 2 | 2.11 |
| CRP level | 1 | 1.05 |
| Age | 0 | 0 |
| Sex | 0 | 0 |
| Distribution of involved small joints hands/feet | 0 | 0 |
| Symmetry of involved joints | 0 | 0 |
| Upper or upper and lower extremities | 0 | 0 |
| TJC68 | 0 | 0 |
| SJC66 | 0 | 0 |
| Rheumatoid factor positive | 0 | 0 |
| Anti-CCP antibodies positive | 0 | 0 |

B. Ha, 2012, year 1 outcome

| Final predictor | n_miss | percent |
| --- | --- | --- |
| Erosive change in baseline X-ray | 3 | 3.16 |
| Morning stiffness duration | 2 | 2.11 |
| CRP level ≥0.5 mg/dL | 1 | 1.05 |
| TJC ≥4 | 0 | 0 |
| SJC ≥4 | 0 | 0 |
| RF positive | 0 | 0 |
| Anti-CCP antibodies positive | 0 | 0 |

Total score was calculated by imputing 0 for all missing values.

C. Rakieh, 2015, PD model, year 2 outcome

| Final predictor | n_miss | percent |
| --- | --- | --- |
| Early morning stiffness duration ≥30 min | 2 | 2.17 |
| Tenderness of small joints | 0 | 0 |
| High level RF and/or anti-CCP | 0 | 0 |
| Power Doppler present | 0 | 0 |

Total score was calculated by imputing 0 for all missing values.

D. Rakieh, 2015, SE model, year 2 outcome

| Final predictor | n_miss | percent |
| --- | --- | --- |
| Shared epitope present | 7 | 7.61 |
| Early morning stiffness duration ≥30 min | 2 | 2.17 |
| Tenderness of small joints | 0 | 0 |
| High level RF and/or anti-CCP | 0 | 0 |

Total score was calculated by imputing 0 for all missing values.

E. Rakieh, 2015, PD+SE model, year 2 outcome

| Final predictor | n_miss | percent |
| --- | --- | --- |
| Shared epitope present | 7 | 7.61 |
| Early morning stiffness duration ≥30 min | 2 | 2.17 |
| Tenderness of small joints | 0 | 0 |
| High level RF and/or anti-CCP | 0 | 0 |
| Power Doppler present | 0 | 0 |

Total score was calculated by imputing 0 for all missing values.

F. Rakieh, 2015, nonPD/nonSE model, year 2 outcome

| Final predictor | n_miss | percent |
| --- | --- | --- |
| Early morning stiffness duration ≥30 min | 2 | 2.17 |
| Tenderness of small joints | 0 | 0 |
| High level RF and/or anti-CCP | 0 | 0 |

Total score was calculated by imputing 0 for all missing values.

G. Ghosh, 2016, year 1 outcome

| Final predictor | n_miss | percent |
| --- | --- | --- |
| Morning stiffness duration* | 2 | 2.11 |
| TJC44 | 0 | 0 |
| Duration of arthritis | 0 | 0 |

*Categorical variable morning stiffness duration (0, 0-30mins, 30mins-1hr, 1-2hrs, 3-4hrs, 4hrs-All day, All day) in the validation dataset was transformed to continuous variable using midpoints (0, 15, 45, 90, 150, 210, 480, 720). Mean imputation was applied for 2 missing values.

H. Di Matteo, 2020, year 1 and year 3 outcome

Linear predictor was calculated by imputing 0 for a missing variable which were also month 6 values.

| Final predictor | n_miss | percent |
| --- | --- | --- |
| Presence of bone erosions in MTP5 joints | 1 | 1.05 |
| High titre anti-CCP2 antibody | 0 | 0 |
| RF positivity | 0 | 0 |
| Smoking exposure (current or previous) | 0 | 0 |

I. Van Boheemen, 2023, combined group, year 2 outcome

There were 25/92 (27.2%) participants with all 4 predictors missing and therefore, excluded. Total score was calculated by imputing 0 for all missing values.

| Final predictor | n_miss | percent |
| --- | --- | --- |
| Over the past month how many days have you had tingling sensations? | 2 | 2.99 |
| Over the past month how many days have you had fatigue? | 2 | 2.99 |
| Over the past month how many days have you had swelling in your joints? | 1 | 1.49 |
| Does your joint pain move from one side of the body to the other? | 0 | 0 |

J. Van Boheemen, 2023, seropositive subgroup, year 2 outcome

There were 25/92 (27.2%) participants with all 3 predictors missing and therefore, excluded. Total score was calculated by imputing 0 for all missing values.

| Final predictor | n_miss | percent |
| --- | --- | --- |
| Over the past month how many days have you had tingling sensations? | 2 | 2.99 |
| Over the past month how many days have you had fatigue? | 2 | 2.99 |
| Over the past month how many days have you had swelling in your joints? | 1 | 1.49 |

K. Duquenne, 2023, simple score, year 1 outcome

| Final predictor | n_miss | percent |
| --- | --- | --- |
| Early morning stiffness duration ≥30 min | 2 | 2.11 |
| Anti-CCP2 value/ULN ≥3 to <10 | 0 | 0 |
| RF positive (> ULN) | 0 | 0 |
| ESR positive (> ULN) | 0 | 0 |

Stiffness variable was imputed using month 3 data.

L. Duquenne, 2023, comprehensive categorical score, year 1 outcome

| Final predictor | n_miss | percent |
| --- | --- | --- |
| US tenosynovitis tendon count* ≥1 | 14 | 14.7 |
| Shared epitope ≥1 allele | 7 | 7.37 |
| Early morning stiffness duration ≥30 min | 2 | 2.11 |
| Age ≥50 years | 0 | 0 |
| Ever-smoker | 0 | 0 |
| Anti-CCP2 value/ULN ≥3 to <10 | 0 | 0 |
| RF positive (> ULN) | 0 | 0 |
| ESR positive (> ULN) | 0 | 0 |
| VAS global pain ≥25 mm | 0 | 0 |
| HAQ ≥0.5 | 0 | 0 |
| US Power Doppler joint count* ≥1 | 0 | 0 |
| US erosion joint count* ≥1 | 0 | 0 |

Stiffness variable was imputed using month 3 data. Total score was calculated by imputing 0 for all missing values.

*partially measured using joints: TSV in 4 sites: extensor carpi ulnaris, finger flexor tendon; Power Doppler in 12 sites: MCP1-3, PIP2, PIP3, wrist (maximum score for Wrist Radiocarpal/Intercarpal/Ulnarcarpal); Bone erosion in 6 sites: MCP2, MTP5, wrist (maximum score for Wrist Radiocarpal/Intercarpal/Ulnarcarpal). Sensitivity analysis in development data using joints available in the validation dataset confirmed minimal impact of missing joints. At 1 year 1 participant who was above the 10% threshold moved to below the 10% threshold.

M. Duquenne, 2023, comprehensive categorical score, year 5 outcome

| Final predictor | n_miss | percent |
| --- | --- | --- |
| US tenosynovitis tendon count* ≥1 | 11 | 14.3 |
| Shared epitope ≥1 allele | 3 | 3.90 |
| Early morning stiffness duration ≥30 min | 1 | 1.30 |
| Age ≥50 years | 0 | 0 |
| Ever-smoker | 0 | 0 |
| Anti-CCP2 value/ULN ≥3 to <10 | 0 | 0 |
| RF positive (> ULN) | 0 | 0 |
| ESR positive (> ULN) | 0 | 0 |
| VAS global pain ≥25 mm | 0 | 0 |
| HAQ ≥0.5 | 0 | 0 |
| US Power Doppler joint count* ≥1 | 0 | 0 |
| US erosion joint count* ≥1 | 0 | 0 |

Stiffness variable was imputed using month 3 data. Total score was calculated by imputing 0 for all missing values.

*partially measured using joints: TSV in 4 sites: extensor carpi ulnaris, finger flexor tendon; Power Doppler in 12 sites: MCP1-3, PIP2, PIP3, wrist (maximum score for Wrist Radiocarpal/Intercarpal/Ulnarcarpal); Bone erosion in 6 sites: MCP2, MTP5, wrist (maximum score for Wrist Radiocarpal/Intercarpal/Ulnarcarpal). Sensitivity analysis in development data using joints available in the validation dataset confirmed minimal impact of missing joints. At 5 years 6 participants who were above the 50% threshold move to below the 10% threshold.

N. Duquenne, 2023, comprehensive continuous score, year 1 outcome

| Final predictor | n_miss | percent |
| --- | --- | --- |
| US abnormalities tenosynovitis* | 10 | 10.5 |
| Shared epitope presence | 7 | 7.37 |
| Alcohol units (per week) | 2 | 2.11 |
| Early morning stiffness duration (0-720 minutes) | 2 | 2.11 |
| CRP | 1 | 1.05 |
| Ever-smoker | 0 | 0 |
| Anti-CCP2 antibody (titre) | 0 | 0 |
| RF (titre) | 0 | 0 |
| ESR | 0 | 0 |
| HAQ (scale 0-3) | 0 | 0 |
| Visual analogue scale for general pain (0-100 mm) | 0 | 0 |
| TJC (0-34) | 0 | 0 |
| US abnormalities Power Doppler* | 0 | 0 |
| US abnormalities bone erosion* | 0 | 0 |

Multiple imputation of missing values.

*partially measured using joints: TSV in 4 sites: extensor carpi ulnaris, finger flexor tendon; Power Doppler in 12 sites: MCP1-3, PIP2, PIP3, wrist (maximum score for Wrist Radiocarpal/Intercarpal/Ulnarcarpal); Bone erosion in 6 sites: MCP2, MTP5, wrist (maximum score for Wrist Radiocarpal/Intercarpal/Ulnarcarpal).

O. Duquenne, 2023, comprehensive continuous score, year 5 outcome

| Final predictor | n_miss | percent |
| --- | --- | --- |
| US abnormalities tenosynovitis* | 8 | 10.4 |
| Shared epitope presence | 3 | 3.90 |
| Alcohol units (per week) | 2 | 2.60 |
| CRP | 1 | 1.30 |
| Early morning stiffness duration (0-720 minutes) | 1 | 1.30 |
| Ever-smoker | 0 | 0 |
| Anti-CCP2 antibody (titre) | 0 | 0 |
| RF (titre) | 0 | 0 |
| ESR | 0 | 0 |
| HAQ (scale 0-3) | 0 | 0 |
| VAS for general pain (0-100 mm) | 0 | 0 |
| TJC (0-34) | 0 | 0 |
| US abnormalities Power Doppler* | 0 | 0 |
| US abnormalities bone erosion* | 0 | 0 |

Multiple imputation of missing values.

*partially measured using joints: TSV in 4 sites: extensor carpi ulnaris, finger flexor tendon; Power Doppler in 12 sites: MCP1-3, PIP2, PIP3, wrist (maximum score for Wrist Radiocarpal/Intercarpal/Ulnarcarpal); Bone erosion in 6 sites: MCP2, MTP5, wrist (maximum score for Wrist Radiocarpal/Intercarpal/Ulnarcarpal).

Anti-CCP=Anti-Cyclic Citrullinated Peptide; CRP=C-Reactive Protein; ESR=Erythrocyte Sedimentation Rate; HAQ=Health assessment questionnaire; RF=Rheumatoid Factor; SJC=Swollen Joint Count; TJC=Tender Joint Count; Ultrasound=US; VAS=Visual Analogue Scale.

Figure S4. Distribution of prediction scores and calibration plots using the independent APIPPRA placebo validation cohort

A. Van der Helm-van Mil, 2008, year 1 outcome

|  | **Non-RA (N=66)** | **RA (N=29)** | **Total (N=95)** |
| --- | --- | --- | --- |
| **Prediction score** |  |  |  |
| Mean (SD) | 6.35 (1.67) | 7.18 (1.64) | 6.60 (1.69) |
| Median [Min, Max] | 5.72 [3.85, 10.2] | 6.84 [4.66, 10.1] | 6.27 [3.85, 10.2] |
| **Prediction score, categorised*** |  |  |  |
| 0-3 | 0 (0%) | 0 (0%) | 0 (0%) |
| 4 | 5 (7.6%) | 0 (0%) | 5 (5.3%) |
| 5 | 18 (27.3%) | 6 (20.7%) | 24 (25.3%) |
| 6 | 17 (25.8%) | 5 (17.2%) | 22 (23.2%) |
| 7 | 8 (12.1%) | 6 (20.7%) | 14 (14.7%) |
| 8 | 11 (16.7%) | 4 (13.8%) | 15 (15.8%) |
| 9 | 2 (3.0%) | 5 (17.2%) | 7 (7.4%) |
| 10 | 5 (7.6%) | 3 (10.3%) | 8 (8.4%) |
| 11-13 | 0 (0%) | 0 (0%) | 0 (0%) |
| **Categorised risk groups** |  |  |  |
| Low (score ≤6) | 36 (54.5%) | 8 (27.6%) | 44 (46.3%) |
| Intermediate (6< score <8) | 17 (25.8%) | 10 (34.5%) | 27 (28.4%) |
| High (score ≥8) | 13 (19.7%) | 11 (37.9%) | 24 (25.3%) |

* Rounded to the nearest number ending .5 or .0

B. Ha, 2012, Simplified prediction model, year 1 outcome

B1

|  | **Non-RA (N=66)** | **RA (N=29)** | **Total (N=95)** |
| --- | --- | --- | --- |
| **Prediction score** |  |  |  |
| Mean (SD) | 5.25 (1.27) | 5.38 (1.30) | 5.29 (1.27) |
| Median [Min, Max] | 5.50 [3.00, 8.50] | 5.50 [3.00, 7.50] | 5.50 [3.00, 8.50] |
| **Prediction score breakdown** |  |  |  |
| ≤2.5 | 0 (0%) | 0 (0%) | 0 (0%) |
| 3 | 4 (6.1%) | 1 (3.4%) | 5 (5.3%) |
| 3.5 | 0 (0%) | 0 (0%) | 0 (0%) |
| 4 | 18 (27.3%) | 9 (31.0%) | 27 (28.4%) |
| 4.5 | 1 (1.5%) | 0 (0%) | 1 (1.1%) |
| 5 | 9 (13.6%) | 4 (13.8%) | 13 (13.7%) |
| 5.5 | 12 (18.2%) | 2 (6.9%) | 14 (14.7%) |
| 6 | 6 (9.1%) | 4 (13.8%) | 10 (10.5%) |
| 6.5 | 10 (15.2%) | 5 (17.2%) | 15 (15.8%) |
| 7 | 0 (0%) | 1 (3.4%) | 1 (1.1%) |
| 7.5 | 5 (7.6%) | 3 (10.3%) | 8 (8.4%) |
| 8 | 0 (0%) | 0 (0%) | 0 (0%) |
| 8.5 | 1 (1.5%) | 0 (0%) | 1 (1.1%) |
| ≥9 | 0 (0%) | 0 (0%) | 0 (0%) |
| **Risk category** |  |  |  |
| Low/Moderate (score <4.5) | 22 (33.3%) | 10 (34.5%) | 32 (33.7%) |
| High (score ≥4.5) | 44 (66.7%) | 19 (65.5%) | 63 (66.3%) |

B2

Predicted probability

Observed proportion

C. Rakieh, 2015, nonPD/nonSE model, year 2 outcome

C1

|  | **Non-RA (N=55)** | **RA (N=37)** | **Total (N=92)** |
| --- | --- | --- | --- |
| **Prediction score** |  |  |  |
| Mean (SD) | 2.85 (0.85) | 3.00 (0.85) | 2.91 (0.85) |
| Median [Min, Max] | 3.00 [1.00, 4.00] | 3.00 [1.00, 4.00] | 3.00 [1.00, 4.00] |
| **Prediction score** |  |  |  |
| 0 | 0 (0%) | 0 (0%) | 0 (0%) |
| 1 | 1 (1.8%) | 2 (5.4%) | 3 (3.3%) |
| 2 | 21 (38.2%) | 7 (18.9%) | 28 (30.4%) |
| 3 | 18 (32.7%) | 17 (45.9%) | 35 (38.0%) |
| 4 | 15 (27.3%) | 11 (29.7%) | 26 (28.3%) |
| **Risk category** |  |  |  |
| Low (score 0) | 0 (0%) | 0 (0%) | 0 (0%) |
| Moderate (score 1-2) | 22 (40.0%) | 9 (24.3%) | 31 (33.7%) |
| High (score 3-4) | 33 (60.0%) | 28 (75.7%) | 61 (66.3%) |

C2

Predicted probability

Observed proportion

D. Rakieh, 2015, PD model, year 2 outcome

D1

|  | **Non-RA (N=55)** | **RA (N=37)** | **Total (N=92)** |
| --- | --- | --- | --- |
| **Prediction score** |  |  |  |
| Mean (SD) | 3.07 (0.96) | 3.41 (1.07) | 3.21 (1.01) |
| Median [Min, Max] | 3.00 [1.00, 5.00] | 3.00 [1.00, 5.00] | 3.00 [1.00, 5.00] |
| **Prediction score** |  |  |  |
| 0 | 0 (0%) | 0 (0%) | 0 (0%) |
| 1 | 1 (1.8%) | 2 (5.4%) | 3 (3.3%) |
| 2 | 17 (30.9%) | 4 (10.8%) | 21 (22.8%) |
| 3 | 17 (30.9%) | 14 (37.8%) | 31 (33.7%) |
| 4 | 17 (30.9%) | 11 (29.7%) | 28 (30.4%) |
| 5 | 3 (5.5%) | 6 (16.2%) | 9 (9.8%) |
| **Risk category** |  |  |  |
| Low (score 0) | 0 (0%) | 0 (0%) | 0 (0%) |
| Moderate (score 1-2) | 18 (32.7%) | 6 (16.2%) | 24 (26.1%) |
| High (score 3-5) | 37 (67.3%) | 31 (83.8%) | 68 (73.9%) |

D2

Predicted probability

Observed proportion

E. Rakieh, 2015, SE model, year 2 outcome

E1

|  | **Non-RA (N=55)** | **RA (N=37)** | **Total (N=92)** |
| --- | --- | --- | --- |
| **Prediction score** |  |  |  |
| Mean (SD) | 2.58 (1.01) | 2.81 (0.88) | 2.67 (0.96) |
| Median [Min, Max] | 3.00 [1.00, 4.00] | 3.00 [1.00, 4.00] | 3.00 [1.00, 4.00] |
| **Prediction score** |  |  |  |
| 0 | 0 (0%) | 0 (0%) | 0 (0%) |
| 1 | 9 (16.4%) | 2 (5.4%) | 11 (12.0%) |
| 2 | 17 (30.9%) | 12 (32.4%) | 29 (31.5%) |
| 3 | 17 (30.9%) | 14 (37.8%) | 31 (33.7%) |
| 4 | 12 (21.8%) | 9 (24.3%) | 21 (22.8%) |
| **Risk category** |  |  |  |
| Low (score 0) | 0 (0%) | 0 (0%) | 0 (0%) |
| Moderate (score 1-2) | 26 (47.3%) | 14 (37.8%) | 40 (43.5%) |
| High (score 3-4) | 29 (52.7%) | 23 (62.2%) | 52 (56.5%) |

E2

Predicted probability

Observed proportion

F. Rakieh, 2015, PD+SE model, year 2 outcome

F1

|  | **Non-RA (N=55)** | **RA (N=37)** | **Total (N=92)** |
| --- | --- | --- | --- |
| **Prediction score** |  |  |  |
| Mean (SD) | 2.80 (1.08) | 3.22 (1.06) | 2.97 (1.08) |
| Median [Min, Max] | 3.00 [1.00, 5.00] | 3.00 [1.00, 5.00] | 3.00 [1.00, 5.00] |
| **Prediction score** |  |  |  |
| 0 | 0 (0%) | 0 (0%) | 0 (0%) |
| 1 | 6 (10.9%) | 1 (2.7%) | 7 (7.6%) |
| 2 | 17 (30.9%) | 9 (24.3%) | 26 (28.3%) |
| 3 | 17 (30.9%) | 13 (35.1%) | 30 (32.6%) |
| 4 | 12 (21.8%) | 9 (24.3%) | 21 (22.8%) |
| 5 | 3 (5.5%) | 5 (13.5%) | 8 (8.7%) |
| **Risk category** |  |  |  |
| Low (score 0-1) | 6 (10.9%) | 1 (2.7%) | 7 (7.6%) |
| Moderate (score 2-3) | 34 (61.8%) | 22 (59.5%) | 56 (60.9%) |
| High (score 4-5) | 15 (27.3%) | 14 (37.8%) | 29 (31.5%) |

F2

Predicted probability

Observed proportion

G. Ghosh, 2016, year 1 outcome

G1

|  | **Non-RA (N=66)** | **RA (N=29)** | **Total (N=95)** |
| --- | --- | --- | --- |
| **Linear predictor** |  |  |  |
| Mean (SD) | 2.20 (10.4) | 3.16 (10.5) | 2.50 (10.4) |
| Median [Min, Max] | -1.03 [-2.84, 48.0] | 0.13 [-2.84, 45.3] | -0.86 [-2.84, 48.0] |

G2

Predicted probability

Observed proportion

H. Di Matteo, 2020, year 1 outcome

|  | **Non-RA (N=66)** | **RA (N=29)** | **Total (N=95)** |
| --- | --- | --- | --- |
| **Linear predictor** |  |  |  |
| Mean (SD) | -0.87 (0.63) | -0.69 (0.74) | -0.82 (0.67) |
| Median [Min, Max] | -0.46 [-2.21, 1.19] | -0.46 [-2.03, 2.14] | -0.46 [-2.21, 2.14] |

H1

H2

Predicted probability

Observed proportion

I. Di Matteo, 2020, year 3 outcome

I1

|  | **Non-RA (N=36)** | **RA (N=50)** | **Total (N=86)** |
| --- | --- | --- | --- |
| **Linear predictor** |  |  |  |
| Mean (SD) | -0.72 (0.55) | -0.55 (0.86) | -0.62 (0.74) |
| Median [Min, Max] | -0.27 [-2.08, -0.27] | -0.27 [-2.34, 2.42] | -0.27 [-2.34, 2.42] |

I2

Predicted probability

Observed proportion

J. Van Boheemen, 2023, combined group, year 2 outcome

J1

|  | **Non-RA (N=37)** | **RA (N=30)** | **Total (N=67)** |
| --- | --- | --- | --- |
| **Prediction score** |  |  |  |
| Mean (SD) | 1.84 (1.50) | 2.00 (1.88) | 1.91 (1.67) |
| Median [Min, Max] | 2.00 [0, 5.00] | 2.00 [0, 5.00] | 2.00 [0, 5.00] |
| **Prediction score breakdown** |  |  |  |
| 0 | 8 (21.6%) | 10 (33.3%) | 18 (26.9%) |
| 1 | 8 (21.6%) | 2 (6.7%) | 10 (14.9%) |
| 2 | 12 (32.4 %) | 9 (30.0%) | 21 (31.3%) |
| 3 | 3 (8.1%) | 2 (6.7%) | 5 (7.5%) |
| 4 | 3 (8.1%) | 1 (3.3%) | 4 (6.0%) |
| 5 | 3 (8.1%) | 6 (20.0%) | 9 (13.4%) |
| **Risk category** |  |  |  |
| Low (score=0) | 8 (21.6%) | 10 (33.3%) | 18 (26.9%) |
| Intermediate (score 1-3) | 23 (62.2%) | 13 (43.3%) | 36 (53.7%) |
| High (score 4-7) | 6 (16.2%) | 7 (23.3%) | 13 (19.4%) |

J2

Predicted risk score

Observed proportion

High risk threshold

K. Van Boheemen, 2023, seropositive subgroup, year 2 outcome

K1

|  | **Non-RA (N=37)** | **RA (N=30)** | **Total (N=67)** |
| --- | --- | --- | --- |
| **Prediction score** |  |  |  |
| Mean (SD) | 2.57 (1.46) | 2.33 (1.71) | 2.46 (1.57) |
| Median [Min, Max] | 2.00 [0, 6.00] | 2.00 [0, 6.00] | 2.00 [0, 6.00] |
| **Prediction score breakdown** |  |  |  |
| 0 | 4 (10.8%) | 6 (20.0%) | 10 (14.9%) |
| 1 | 1 (2.7%) | 3 (10.0%) | 4 (6.0%) |
| 2 | 18 (48.6%) | 10 (33.3%) | 28 (41.8%) |
| 3 | 2 (5.4%) | 0 (0%) | 2 (3.0%) |
| 4 | 9 (24.3%) | 9 (30.0%) | 18 (26.9%) |
| 5 | 2 (5.4%) | 1 (3.3%) | 3 (4.5%) |
| 6 | 1 (2.7%) | 1 (3.3%) | 2 (3.0%) |
| **Risk category** |  |  |  |
| Low (score 0-2) | 23 (62.2%) | 19 (63.3%) | 42 (62.7%) |
| High (score 3-6) | 14 (37.8%) | 11 (36.7%) | 25 (37.3%) |

K2

Predicted risk score

Observed proportion

High risk threshold

L. Duquenne, 2023, simple score, year 1 outcome

L1

|  | **Non-RA (N=66)** | **RA (N=29)** | **Total (N=95)** |
| --- | --- | --- | --- |
| **Prediction score** |  |  |  |
| Mean (SD) | 29.1 (7.51) | 32.2 (6.41) | 30.0 (7.30) |
| Median [Min, Max] | 28.0 [11.0, 43.0] | 34.0 [20.0, 43.0] | 28.0 [11.0, 43.0] |
| **Prediction score breakdown** |  |  |  |
| ≤10 | 0 (0%) | 0 (0%) | 0 (0%) |
| 11 | 2 (3.0%) | 0 (0%) | 2 (2.1%) |
| 12-16 | 0 (0%) | 0 (0%) | 0 (0%) |
| 17 | 5 (7.6%) | 0 (0%) | 5 (5.3%) |
| 18 | 0 (0%) | 0 (0%) | 0 (0%) |
| 19 | 4 (6.1%) | 0 (0%) | 4 (4.2%) |
| 20 | 0 (0%) | 2 (6.9%) | 2 (2.1%) |
| 21-22 | 0 (0%) | 0 (0%) | 0 (0%) |
| 23 | 4 (6.1%) | 0 (0%) | 4 (4.2%) |
| 24-25 | 0 (0%) | 0 (0%) | 0 (0%) |
| 26 | 2 (3.0%) | 2 (6.9%) | 4 (4.2%) |
| 27 | 0 (0%) | 0 (0%) | 0 (0%) |
| 28 | 22 (33.3%) | 10 (34.5%) | 32 (33.7%) |
| 29-31 | 0 (0%) | 0 (0%) | 0 (0%) |
| 32 | 3 (4.5%) | 0 (0%) | 3 (3.2%) |
| 33 | 0 (0%) | 0 (0%) | 0 (0%) |
| 34 | 13 (19.7%) | 6 (20.7%) | 19 (20.0%) |
| 35-36 | 0 (0%) | 0 (0%) | 0 (0%) |
| 37 | 6 (9.1%) | 5 (17.2%) | 11 (11.6%) |
| 38-42 | 0 (0%) | 0 (0%) | 0 (0%) |
| 43 | 5 (7.6%) | 4 (13.8%) | 9 (9.5%) |
| **Risk groups for referral to secondary care** |  |  |  |
| Low (score <18) | 7 (10.6%) | 0 (0%) | 7 (7.4%) |
| High (score ≥18) | 59 (89.4%) | 29 (100%) | 88 (92.6%) |

L2

Observed proportion

Predicted probability

M. Duquenne, 2023, comprehensive categorical score, year 1 outcome

|  | **Non-RA (N=66)** | **RA (N=29)** | **Total (N=95)** |
| --- | --- | --- | --- |
| **Prediction score** |  |  |  |
| Mean (SD) | 23.5 (5.54) | 26.2 (5.26) | 24.3 (5.57) |
| Median [Min, Max] | 24.0 [9.00, 36.0] | 26.0 [16.0, 36.0] | 24.0 [9.00, 36.0] |
| **Prediction score breakdown** |  |  |  |
| ≤8 | 0 (0%) | 0 (0%) | 0 (0%) |
| 9 | 1 (1.5%) | 0 (0%) | 1 (1.1%) |
| 13 | 1 (1.5%) | 0 (0%) | 1 (1.1%) |
| 14 | 2 (3.0%) | 0 (0%) | 2 (2.1%) |
| 16 | 5 (7.6%) | 1 (3.4%) | 6 (6.3%) |
| 17 | 3 (4.5%) | 2 (6.9%) | 5 (5.3%) |
| 18 | 1 (1.5%) | 0 (0%) | 1 (1.1%) |
| 19 | 4 (6.1%) | 1 (3.4%) | 5 (5.3%) |
| 21 | 4 (6.1%) | 0 (0%) | 4 (4.2%) |
| 22 | 4 (6.1%) | 2 (6.9%) | 6 (6.3%) |
| 23 | 4 (6.1%) | 2 (6.9%) | 6 (6.3%) |
| 24 | 9 (13.6%) | 3 (10.3%) | 12 (12.6%) |
| 25 | 4 (6.1%) | 2 (6.9%) | 6 (6.3%) |
| 26 | 6 (9.1%) | 2 (6.9%) | 8 (8.4%) |
| 27 | 5 (7.6%) | 3 (10.3%) | 8 (8.4%) |
| 28 | 1 (1.5%) | 2 (6.9%) | 3 (3.2%) |
| 29 | 1 (1.5%) | 0 (0%) | 1 (1.1%) |
| 30 | 3 (4.5%) | 3 (10.3%) | 6 (6.3%) |
| 31 | 3 (4.5%) | 2 (6.9%) | 5 (5.3%) |
| 32 | 2 (3.0%) | 0 (0%) | 2 (2.1%) |
| 33 | 2 (3.0%) | 0 (0%) | 2 (2.1%) |
| 34 | 0 (0%) | 3 (10.3%) | 3 (3.2%) |
| 36 | 1 (1.5%) | 1 (3.4%) | 2 (2.1%) |
| 37-42 | 0 (0%) | 0 (0%) | 0 (0%) |
| **Risk groups for frequent follow up** |  |  |  |
| Low (score <15) | 4 (6.1%) | 0 (0%) | 4 (4.2%) |
| High (score ≥15) | 62 (93.9%) | 29 (100%) | 91 (95.8%) |

N. Duquenne, 2023, comprehensive categorical score, year 5 outcome

N1

|  | **Non-RA (N=19)** | **RA (N=58)** | **Total (N=77)** |
| --- | --- | --- | --- |
| **Prediction score** |  |  |  |
| Mean (SD) | 22.6 (4.87) | 25.6 (5.26) | 24.8 (5.30) |
| Median [Min, Max] | 24.0 [14.0, 30.0] | 25.5 [13.0, 36.0] | 25.0 [13.0, 36.0] |
| **Prediction score breakdown** |  |  |  |
| ≤12 | 0 (0%) | 0 (0%) | 0 (0%) |
| 13 | 0 (0%) | 1 (1.7%) | 1 (1.3%) |
| 14 | 2 (10.5%) | 0 (0%) | 2 (2.6%) |
| 16 | 1 (5.3%) | 2 (3.4%) | 3 (3.9%) |
| 17 | 1 (5.3%) | 3 (5.2%) | 4 (5.2%) |
| 19 | 2 (10.5%) | 2 (3.4%) | 4 (5.2%) |
| 21 | 0 (0%) | 4 (6.9%) | 4 (5.2%) |
| 22 | 2 (10.5%) | 3 (5.2%) | 5 (6.5%) |
| 23 | 1 (5.3%) | 4 (6.9%) | 5 (6.5%) |
| 24 | 3 (15.8%) | 7 (12.1%) | 10 (13.0%) |
| 25 | 1 (5.3%) | 3 (5.2%) | 4 (5.2%) |
| 26 | 1 (5.3%) | 5 (8.6%) | 6 (7.8%) |
| 27 | 3 (15.8%) | 4 (6.9%) | 7 (9.1%) |
| 28 | 0 (0%) | 3 (5.2%) | 3 (3.9%) |
| 29 | 1 (5.3%) | 0 (0%) | 1 (1.3%) |
| 30 | 1 (5.3%) | 6 (10.3%) | 7 (9.1%) |
| 31-36 | 0 (0%) | 11 (19.0%) | 11 (14.3%) |
| 37-42 | 0 (0%) | 0 (0%) | 0 (0%) |
| **Risk groups for frequent follow up and intervention** |  |  |  |
| Low (score <24) | 9 (47.4%) | 19 (32.8%) | 28 (36.4%) |
| High (score ≥24) | 10 (52.6%) | 39 (67.2%) | 49 (63.6%) |

N2

Observed proportion

Predicted probability

O. Duquenne, 2023, comprehensive continuous score, year 1 outcome

O1

|  | **Non-RA (N=66)** | **RA (N=29)** | **Total (N=95)** |
| --- | --- | --- | --- |
| **Linear predictor** |  |  |  |
| Mean (SD) | 0.19 (9.42) | 1.46 (0.53) | 0.58 (7.86) |
| Median [Min, Max] | 1.34 [-75.1, 2.60] | 1.38 [0.53, 2.76] | 1.35 [-75.1, 2.76] |

O2

Predicted probability

Observed proportion

P. Duquenne, 2023, comprehensive continuous score, year 5 outcome

P1

|  | **Non-RA (N=19)** | **RA (N=58)** | **Total (N=77)** |
| --- | --- | --- | --- |
| **Linear predictor** |  |  |  |
| Mean (SD) | 1.32 (0.40) | 1.43 (0.55) | 1.40 (0.52) |
| Median [Min, Max] | 1.30 [0.66, 2.36] | 1.38 [-0.55, 2.76] | 1.35 [-0.55, 2.76] |

P2

Predicted probability

Observed proportion

REFERENCES

1.	Hanley JA, McNeil BJ. The meaning and use of the area under a receiver operating characteristic (ROC) curve. Radiology. 1982;143(1):29-36.

2.	Harrell FE, Jr., Lee KL, Mark DB. Multivariable prognostic models: issues in developing models, evaluating assumptions and adequacy, and measuring and reducing errors. Stat Med. 1996;15(4):361-87.

3.	Riley RD, Ensor J, Snell KI, et al. External validation of clinical prediction models using big datasets from e-health records or IPD meta-analysis: opportunities and challenges. BMJ. 2016;353:i3140.

4.	Brier G. Verification of forecasts expressed in terms of probability. Monthly Weather Review. 1950(78):1–3.

5.	van Geloven N, Giardiello D, Bonneville EF, et al. Validation of prediction models in the presence of competing risks: a guide through modern methods. BMJ. 2022;377:e069249.

6.	Van Calster B, McLernon DJ, van Smeden M, et al. Calibration: the Achilles heel of predictive analytics. BMC Med. 2019;17(1):230.
